# A combined tissue‐engineered/*in silico* signature tool patient stratification in lung cancer

**DOI:** 10.1002/1878-0261.12323

**Published:** 2018-06-22

**Authors:** Claudia Göttlich, Meik Kunz, Cornelia Zapp, Sarah L. Nietzer, Heike Walles, Thomas Dandekar, Gudrun Dandekar

**Affiliations:** ^1^ Tissue Engineering and Regenerative Medicine University Hospital Wuerzburg Germany; ^2^ Fraunhofer Institute for Silicate Research (ISC) Translational Center Regenerative Therapies Wuerzburg Germany; ^3^ Department of Bioinformatics Biocenter University of Wuerzburg Germany; ^4^ Institute for Pharmaceutics and Molecular Biotechnology (IPMB) University of Heidelberg Germany; ^5^ Structural and Computational Biology EMBL Heidelberg Germany

**Keywords:** 3D lung tumor model, Boolean signaling network, chemoresistance, HSP90 inhibitor, *in silico* drug screening tool, *KRAS* mutation signature

## Abstract

Patient‐tailored therapy based on tumor drivers is promising for lung cancer treatment. For this, we combined *in vitro* tissue models with *in silico* analyses. Using individual cell lines with specific mutations, we demonstrate a generic and rapid stratification pipeline for targeted tumor therapy. We improve *in vitro* models of tissue conditions by a biological matrix‐based three‐dimensional (3D) tissue culture that allows *in vitro* drug testing: It correctly shows a strong drug response upon gefitinib (Gef) treatment in a cell line harboring an EGFR‐activating mutation (HCC827), but no clear drug response upon treatment with the HSP90 inhibitor 17AAG in two cell lines with *KRAS* mutations (H441, A549). In contrast, 2D testing implies wrongly *KRAS* as a biomarker for HSP90 inhibitor treatment, although this fails in clinical studies. Signaling analysis by phospho‐arrays showed similar effects of EGFR inhibition by Gef in HCC827 cells, under both 2D and 3D conditions. Western blot analysis confirmed that for 3D conditions, HSP90 inhibitor treatment implies different p53 regulation and decreased MET inhibition in HCC827 and H441 cells. Using *in vitro* data (western, phospho‐kinase array, proliferation, and apoptosis), we generated cell line‐specific *in silico* topologies and condition‐specific (2D, 3D) simulations of signaling correctly mirroring *in vitro* treatment responses. Networks predict drug targets considering key interactions and individual cell line mutations using the Human Protein Reference Database and the COSMIC database. A signature of potential biomarkers and matching drugs improve stratification and treatment in *KRAS*‐mutated tumors. *In silico* screening and dynamic simulation of drug actions resulted in individual therapeutic suggestions, that is, targeting HIF1A in H441 and LKB1 in A549 cells. In conclusion, our *in vitro* tumor tissue model combined with an *in silico* tool improves drug effect prediction and patient stratification. Our tool is used in our comprehensive cancer center and is made now publicly available for targeted therapy decisions.

**Abbreviations**
17AAG: 17‐*N*‐allylamino‐17‐demethoxygeldanamycin
2D: two‐dimensional
3D: three‐dimensional
ADME: absorption, distribution, metabolism, excretion
AICAR: 5‐aminoimidazole‐4‐carboxamide ribonucleotide
BioVaSc^®^: Biological Vascularized Scaffold
COSMIC: Catalogue Of Somatic Mutations In Cancer
DRPs: differentially regulated proteins
DrumPID: Drug‐minded Protein Interaction Database
Gef: gefitinib
HE: hematoxylin and eosin
HPRD: Human Protein Reference Database
HSP: heat shock protein
NGS: next‐generation sequencing
NSCLC: non‐small cell lung cancer
PK: phospho‐kinase
RTK: receptor tyrosine kinase
SMILES: Simplified Molecular Input Line Entry Specification
SQUAD: Stardardized Qualitative Dynamical Modelling Suite
TKI: tyrosine kinase inhibitor

## 1 Introduction

In the highly mortal lung cancer, next‐generation sequencing (NGS) approaches successfully reveal driver mutations to stratify lung cancer patients for targeted therapies (Buettner *et al*., 2013). Tyrosine kinase inhibitor (TKI) treatment shows remarkable response rates, exemplified by EGFR inhibitors in patients with activating *EGFR* mutations (Ciardiello *et al*., 2004; Paez *et al*., 2004; Russo *et al*., 2015). However, often the therapy is only initially successful and then followed by secondary resistance. Unfortunately, tumors with *KRAS* mutations are primarily resistant to targeted therapies and comprise about 30–40% of all patients (Sequist *et al*., 2011).

Due to poor correlations of preclinical *in vitro* data to drug efficacy in patients, particularly in the field of cancer (Bhattacharjee, 2012), new 3D tumor models arise, such as spheroids, microfluidic devices, organoids, and matrix‐based approaches (Alemany‐Ribes and Semino, 2014; Edmondson *et al*., 2014; Xu *et al*., 2014). The generally high proliferation rate in 2D cell cultures is one reason for false‐positive predictions of cytostatic compounds (Cree *et al*., 2010). Decreased proliferation of tumor cells corresponding to clinical specimens was demonstrated on our scaffold (Göttlich *et al*., 2016; Nietzer *et al*., 2016; Stratmann *et al*., 2014) originating from the *Biological Vascularized Scaffold* (BioVaSc^®^) (Linke *et al*., 2007; Schanz *et al*., 2010). It maintains the extracellular matrix, including structures of the basement membrane, enabling physiological anchorage of epithelial cells. Earlier, we combined the tissue‐engineered lung tumor model with its *in silico* representation to investigate tumor and, thereby, drug‐relevant dependencies – also in the context of resistance (Göttlich *et al*., 2016; Stratmann *et al*., 2014).

In this study, we introduce a patient stratification tool according to tumor drivers as a promising decision tool for precision medicine in lung cancer. This is exemplified here by studying individual *in vitro* cell lines and their differing drug responses in 2D and 3D, and by integrating these data in corresponding *in silico* analyses for target predictions. The tool is generic and provides a rapid stratification pipeline that can support tumor boards to utilize more and more clinically available NGS data from individual patients.

We studied how a biological matrix‐based 3D tissue culture allows *in vitro* drug testing of relevant lung cancer subgroups. To unravel signal cascade outputs in more detail, we investigated apoptosis and proliferation as drug responses. Regarding the EGFR inhibition with the TKI gefitinib (Gef) in a cell line carrying the corresponding biomarker, we observed an enhancement in apoptosis induction compared to 2D. Moreover, we exemplified our stratification tool by looking at responses of two further cell lines (A549, H441) harboring *KRAS* mutations to the HSP90 inhibitor 17AAG. In contrast to the EGFR inhibition, in this setting only the 3D system could predict no drug efficiency in line with clinical findings. Therefore, we analyzed differences in signaling changes upon treatment between cell lines and between 2D and 3D conditions. Using the experimental data of the 3D tissue model, we created (a) *in silico* cell line‐specific topologies of the centrally involved proteins including their logical connectivity. Based on these data, (b) dynamic *in silico* simulations mirrored the differences in cellular responses apparent in the experiments. Considering protein neighbors of central important signaling cascades and cell‐specific mutations from databases resulted (c) in larger *in silico* networks which were next screened *in silico* for individual therapeutic options for each cell line. Resulting drug suggestions reflect clinical experiences and include comprehensive FDA‐approved treatment options. In its unique combination, the tool raises hopes of efficiently exploiting upcoming sequence information of patient tumors in the near future for targeted therapy.

## 2 Results

### 2.1 Analysis path

To exemplify the process of how a single patient's sequence could be integrated into our new preclinical prediction tool, we chose three cell lines representing different patient subgroups regarding *KRAS* mutation (HCC827: *KRAS* wild‐type; A549: *KRAS* mutant, independent; H441: *KRAS* mutant, dependent) (Singh *et al*., 2009).

**Figure 1:**
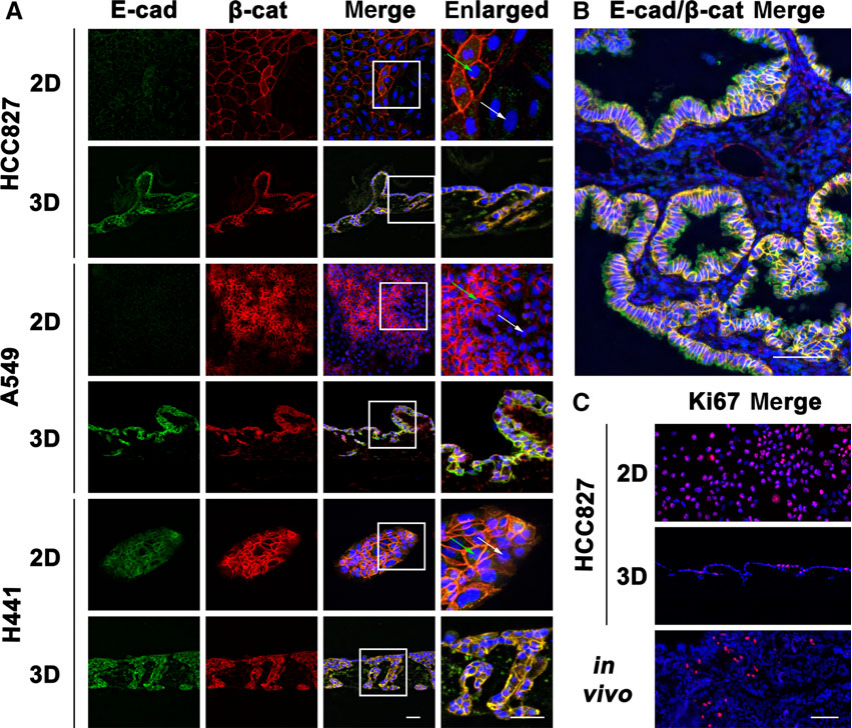
Improved reflection of tumor characteristics by the 3D tissue‐engineered lung tumor model. Tested tumor cell line populations display more homogeneous marker expression in 3D as well as reduced proliferation correlating to tumors. (A) Cells cultured in 2D and 3D conditions shown with immunofluorescence by double stain for E‐cadherin and β‐catenin. Green arrows indicate positive cells and white arrows negative. Scale bars are 50 μm. (B) Paraffin‐embedded adenocarcinoma from patient biopsy was immunofluorescence‐double‐stained against E‐cadherin and β‐catenin. Scale bar is 100 μm. (C) The expression of the proliferation marker Ki67 was detected by immunofluorescence staining of 2D and 3D cultured HCC827 cells, as well as *in vivo* tissue from a patient biopsy. Scale bar is 100 μm.

**Figure 2:**
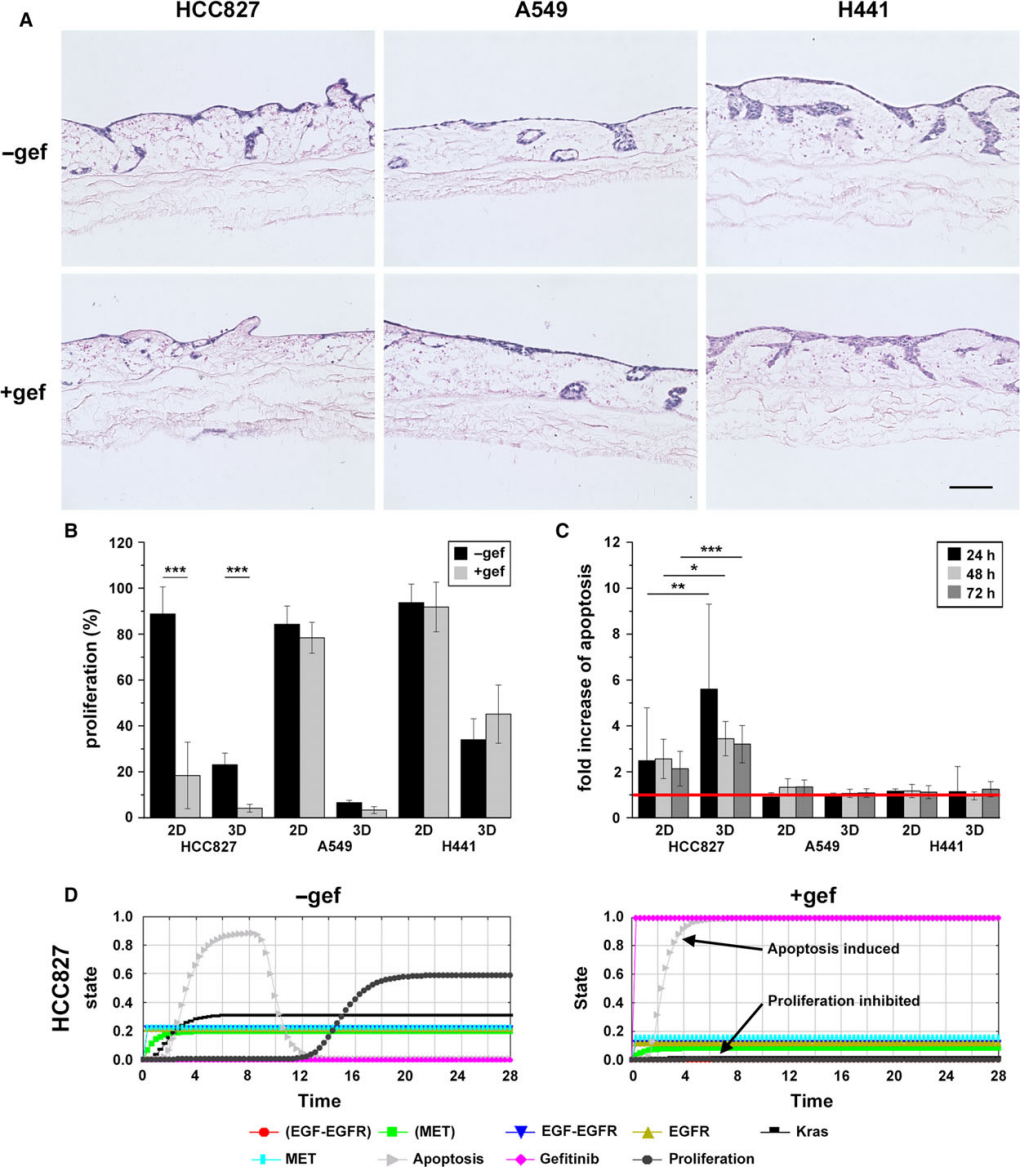
Biomarker‐dependent response upon EGFR inhibition is improved in 3D and can also be simulated *in silico*. (A) Cells cultured in 3D that were either treated with 1 μm Gef or used as untreated controls were paraffin‐embedded and HE‐stained. Scale bar is 100 μm. (B) The proliferation rate (proliferative cells per total cell number) was determined by counting Ki67‐positive cells from immunofluorescence staining in 10 images per sample. Total cell number was quantified by DAPI counterstaining. ***P < 0.001, *n* ≥ 4. (C) Apoptosis was investigated by M30 CytoDeath™ ELISA. Therefore, supernatants of treated and untreated samples were collected prior to and at 24, 48, and 72 h after treatment. Concentrations of M30 in samples after treatment were normalized to T0 values from samples taken before treatment and related to untreated samples (red line). ***P < 0.001, *n* ≥ 4. (D) *In silico* simulation of the Gef treatment (right, pink curve full on at 1.0) shows reduced proliferation (right, black curve) only in HCC827 cells and higher apoptosis (right, gray curve), as compared to untreated cells (left, pink curve switched off at 0.0). Figure S2A shows the *in silico* topology and Fig. S2B the simulations for A549 and H441. *P < 0.05, **P < 0.01

**Figure 3:**
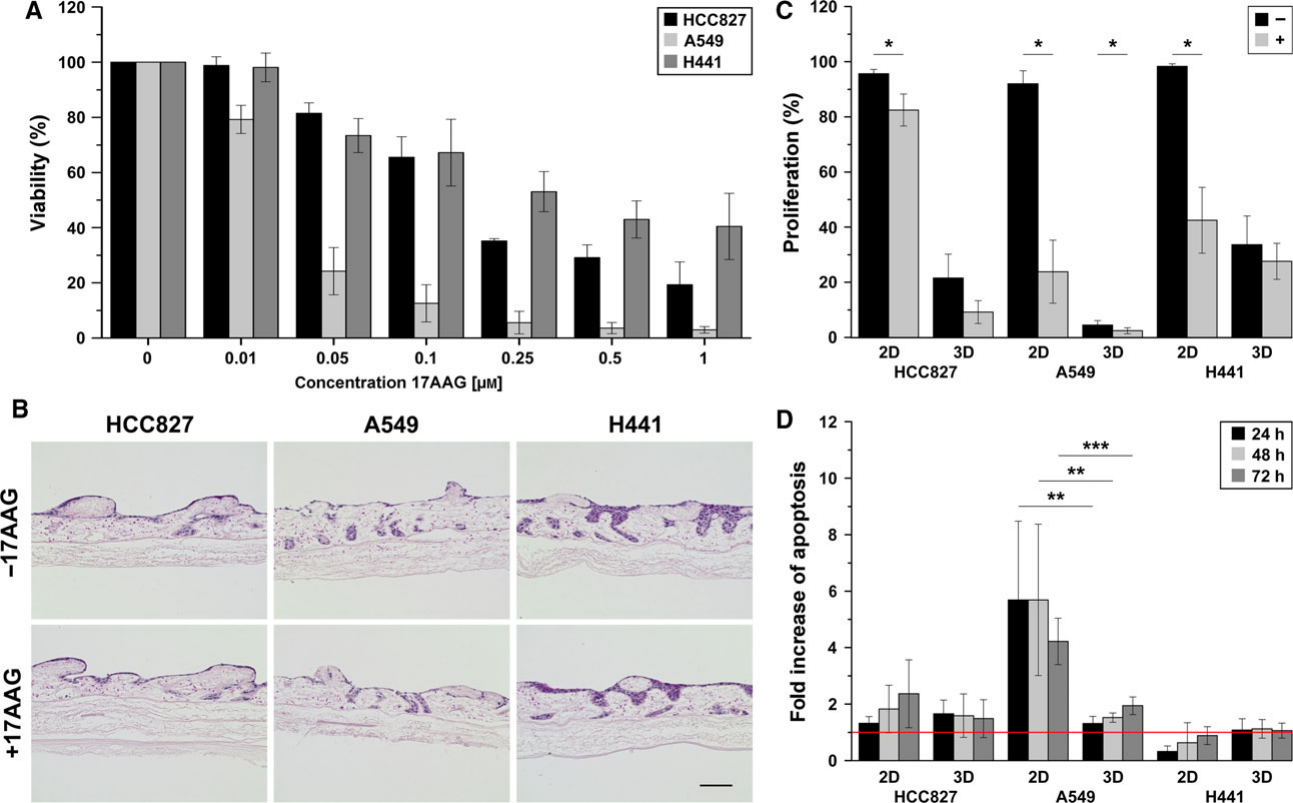
Effects of the HSP90 inhibitor 17AAG diminish in 3D and cannot be aligned to the biomarker KRAS (A549/H441). Strong treatment responses regarding viability, proliferation, and apoptosis can be observed only in 2D conditions. (A) Cells cultured in 2D conditions were treated with different concentrations of the HSP90 inhibitor 17AAG. Viability was determined after 3 days of treatment by a CellTiter‐Glo^®^ Luminescent Cell Viability Assay. *n* ≥ 4. (B) 3D cultured cells were treated with 0.25 μm 17AAG, paraffin‐embedded, and HE‐stained. Scale bar is 100 μm. (C) The proliferation rate in 2D and 3D was determined by counting Ki67‐positive cells from immunofluorescence staining in 10 images per sample. Total cell number was quantified by DAPI counterstaining. *P < 0.05, *n* ≥ 4. (D) Apoptosis was investigated by M30 CytoDeath™ ELISA. Therefore, supernatants of treated and untreated samples were collected prior to and at 24, 48, and 72 h after treatment. Concentrations of M30 in samples after treatment were normalized to T0 values from samples taken before treatment and related to untreated samples (red line). *P < 0.05, ***P < 0.001, *n* ≥ 4. **P < 0.01.

**Figure 4:**
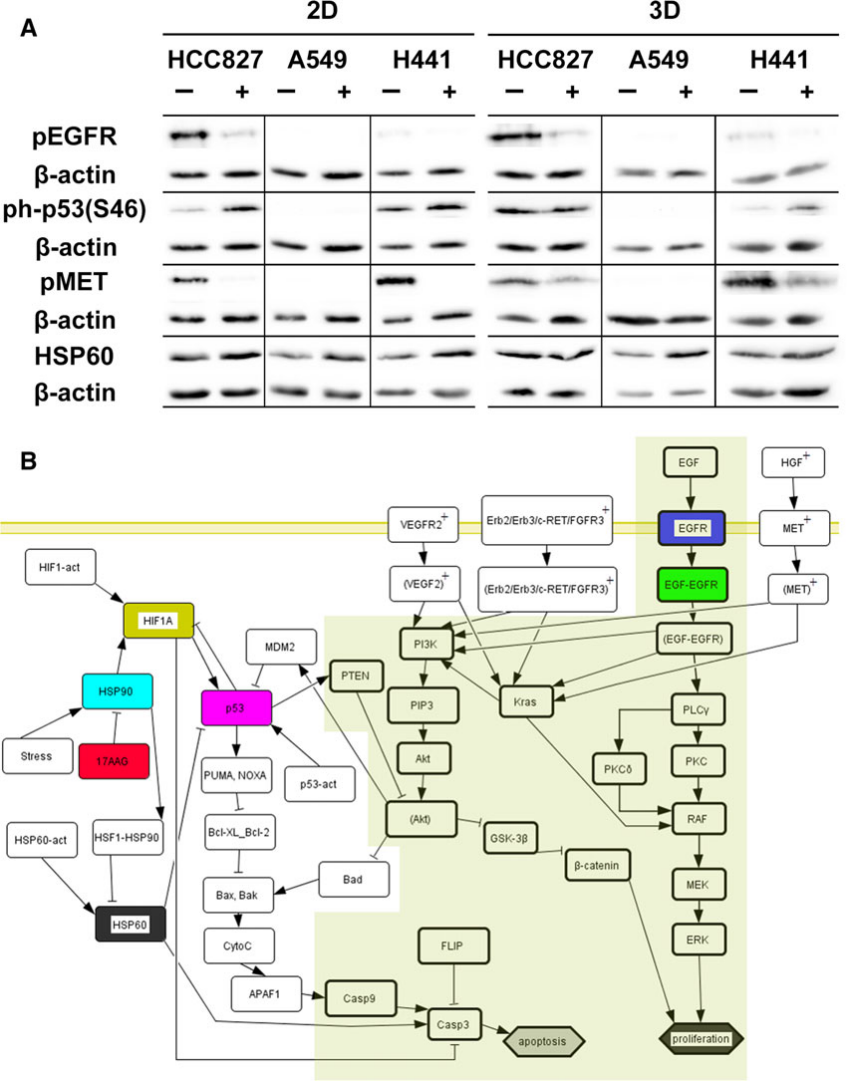
Signaling changes after HSP90 inhibition differ between 2D and 3D and between the different cell lines and are integrated into *in silico* topologies. (A) Cells cultured in 2D and 3D were treated with 0.25 μm 17AAG for 24 h (2D) or 72 h (3D). The signaling changes of different phospho‐proteins were analyzed by western blot. The same lysates were used for the pEGFR and ph‐p53(S46) blots of all three cell lines in 2D and 3D HCC827 and for ph‐p53(S46) and pMET blots in 3D H441; thus, the same β‐actin loading control is shown below these phospho‐proteins. (B) DRPs from the *in vitro* 3D system are connected *in silico* to the central tumor signaling cascade. Here, we show the topology shared between all three cell lines. Colors reflect important input (treatment), signaling proteins, and cellular output (proliferation and apoptosis). Proteins (‘nodes’) from the topology of Stratmann *et al*. (2014) are bold rimmed and have an olive background; proteins added specifically to the *in silico* topology are presented as simple boxes; protein node colors are as in the simulation curves; cell line‐specific proteins (‘nodes’) appear as plus (+). Specific topologies and simulation results for each cell line are given in the Supporting information.

To unravel the complex interdependent signaling network in lung cancer in different mutational backgrounds, we experimentally measured, in a global approach using phospho‐arrays signaling, changes to Gef and 17‐allylamino‐17‐demethoxygeldanamycin (17AAG) treatment in our three different cell lines (Figs 1, 2, 3; Table 1; Figs S1–S3; first simulation is in S2; further *in silico* analyses in S4–S7). Firstly, we recognized that with the EGFR inhibitor Gef affected proteins are roughly the same in HCC827 (EGFR mutated) in 2D and 3D models (Table 1A, Fig. S1). By analyzing signaling changes upon the HSP90 inhibitor treatment by phospho‐arrays and western blot in all three cell lines in 2D and in 3D, it became obvious that besides MET, changes between the 2D and the 3D models concern mostly p53 and HSP60 (Table 1B; Figs 4A and S3).

**Table 1:** Comparison of the phosphorylation data showing different regulation between the cell lines in the 2D and 3D system for Gef and 17AAG

	HCC827		A549		H441	
	2D	3D	2D	3D	2D	3D
(A) Gef treatment^a^						
pEGFR	↓	↓	0	0	const.	const.
pErbB2	↓	0	0	0	0	0
pMET	↓	↓	0	0	const.	const.
(B) 17AAG treatment^b^						
pEGFR	↓	↓	0	0	↓↓↓	↓↓
pErbB2	↓	↓	0	0	↓	↓
pErbB3	↓	↓	0	0	↓	↓
pMET	↓↓	↓	0	0	↓↓↓	↓
pc‐Ret	↓	↓	0	0	↓	↓
pVEGFR2	0	0	0	0	↓	↓
pFGFR3	0	0	0	0	0	↓
p‐p53 (S46)	↑↑↑	const.	0	0	const.	↑↑↑
HSP60	const.	const.	↑	↑↑	↑	const.

a Based on the RTK array data, this is a qualitative summary of all proteins measured, showing a phosphorylation difference in at least one cell line upon Gef treatment (0 reflects no activation, and const. means no activation change after treatment). Experimental data are shown in Fig. S1.

b Based on the western blots (semiquantitative, more than one arrow possible) and RTK array data (qualitative, only one arrow possible), this is a summary of all proteins measured, showing a phosphorylation difference in at least one cell line upon 17AAG treatment (detailed experimental data shown in Figs 4A and S3A; 0 reflects no activation, const. means no activation change after treatment).

**Figure 5:**
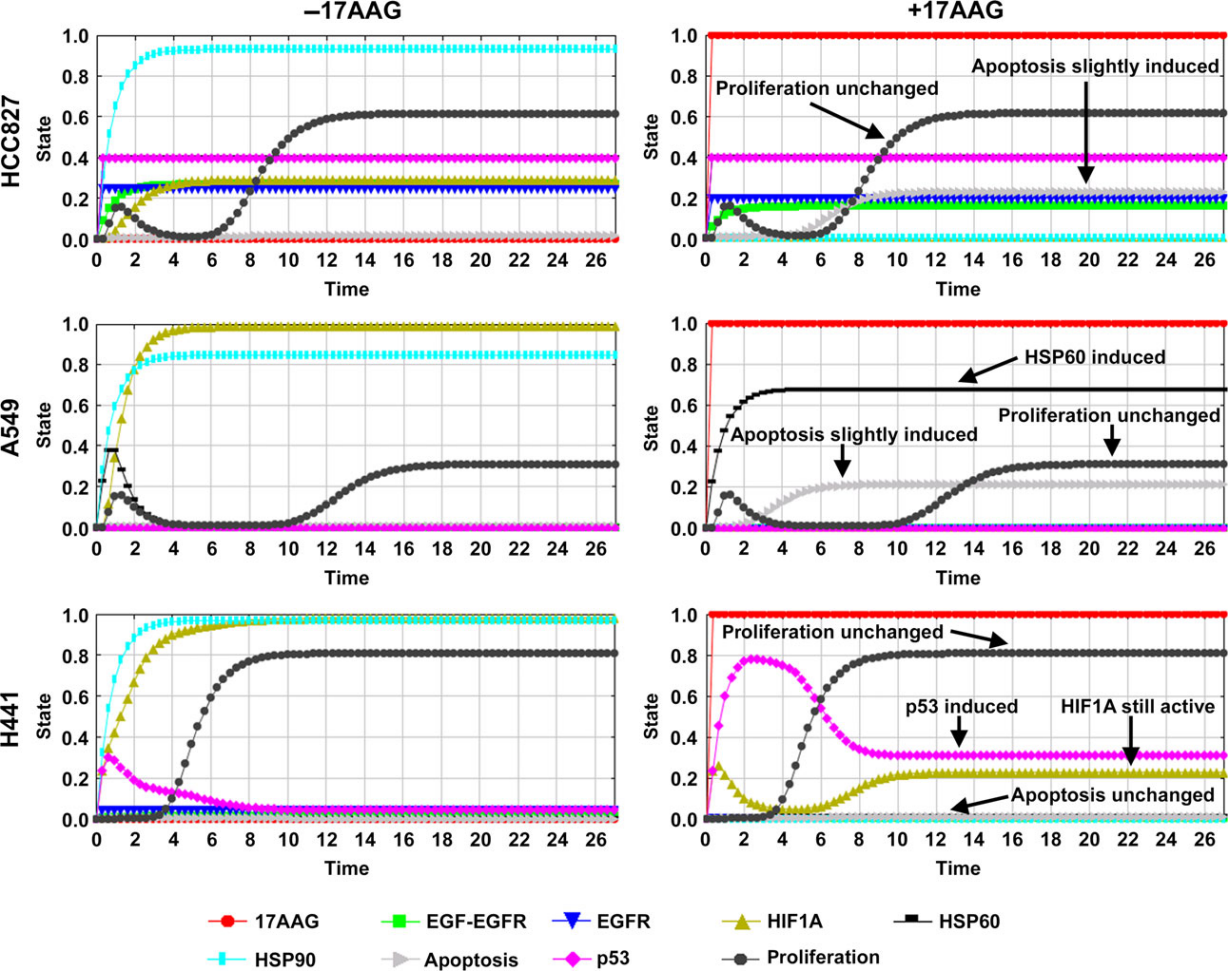
Cell line‐specific *in silico* simulations for 17AAG treatment according to data from the 3D system. Simulations of the 17AAG treatment reflect the *in vitro* data. Coloring of the curves is according to the network node colors shared for all three cell lines shown in Fig. 4B. Cell line‐specific pathway differences included are given in Table 2. Top: Simulation of the 17AAG treatment in HCC827 cells (right, red curve at full activation) results in slightly induced apoptosis (gray curve at 0.2) and unchanged proliferation (black curve), as compared to untreated cells (left, red curve at 0.0, no treatment). Middle: The *in silico* simulation of the 17AAG treatment for A549 shows only low apoptosis induction (0.2); we see no therapeutic effect on proliferation (black curve, dots) compared to untreated cells. However, HSP60 (black curve, squares) is induced after 17AAG treatment, similar to the *in vitro* data. Bottom: In H441 cells, apoptosis is not elevated over time and no effect on proliferation can be obtained. p53 (pink curve) is induced after 17AAG treatment and correlates with the *in vitro* data.

Regarding *in silico* analyses, we first set up cell line‐specific *in silico* topologies by integrating important signaling nodes that distinguished the cell lines upon Gef and 17AAG treatment into our basic *in silico* topology (Table 2; Stratmann *et al*., 2014). The nodes of this basic topology are marked in all newly generated topologies with bold printed borders. After the generation of these cell line‐specific *in silico* topologies, we mirrored the *in vitro* treatment response of Gef and 17AAG, by applying semiquantitative Boolean simulations using the software squad (Stardardized Qualitative Dynamical Modelling Suite). Based on the logical connectivity of each cellular topology, this software models the dynamic evolution of the included signaling cascades using exponential functions (Di Cara *et al*., 2007). Furthermore, different activation strengths for each node of the signaling cascade are considered in the simulations that were necessary to adapt the *in silico* simulation results to the *in vitro* results for differences of 3D and 2D cultures. Input into the topology of Fig. 4B is listed in Table 3A and B for 3D conditions (further network analyses in Tables S1–S3) and in Box S2 for 2D conditions. Simulations’ output of 3D conditions is presented in Figs 2D and S2 for Gef and in Fig. 5 for 17AAG treatment. Simulation results in 2D conditions of KRAS‐mutated cell lines are represented in Fig. S6 for Gef and in Fig. S7 for 17AAG treatment.

**Table 2:** Cell line‐specific proteins introduced in addition to the original topology.^a^

Cell line	Gef	17AAG
HCC827 (3D)	MET cascade^+^	MET cascade^+^; Erb2 cascade^+^;
		Erb3 cascade^+^; c‐RET cascade^+^;
		HSP90; HSP60; HIF1A; p53
A549 (3D)	–	HSP90; HSP60
		HIF1A; p53
H441 (3D)	MET cascade^+^	MET cascade^+^; HSP90; HSP60;
		HIF1A; p53; Erb2 cascade^+^;
		VEGF2 cascade^+^; Erb3
		cascade^+^; c‐RET cascade^+^;
		FGFR3 cascade^+^

a Listed are the proteins extending the network of Stratmann *et al*. (2014) that responds upon Gef or 17AAG treatment and which were measured in arrays and western blots. According to interaction analysis p53, HSP60, HIF1A and HSP90 are added as cascades around 17AAG. A plus (+) indicates cell line‐specific protein nodes added according to the experimental data. Further cell line‐specific protein nodes according to COSMIC and relevant to our *in silico* network as being close to or in our signaling cascades are listed in Table S3 (9 in A549, 18 in H441). Cell‐specific mutations analyzed in detail are shown in Figs 6 and 7. A complete list of all cell line‐specific mutations known is given in Table S2.

**Table 3:** Different activation strengths for each node for *in silico* simulations. Cell line‐specific differences in pathway activities on (A) Gef^a^ and (B) 17AAG^a^ and (C) AMPK activator and HIF1A inhibitor^b^

Cell line	Parameter	(−) gef	(+) gef
(A)			
HCC827 (3D)	MET	0.22	0.15
	EGFR	0.22	0.12
	EGF‐EGFR	0.22	0.12
	FLIP	0.6	0.5
A549 (3D)	KRAS^c^	0.413	0.413
	FLIP	0.6	0.6
H441 (3D)	KRAS^c^	0.43	0.43
	EGFR	0.205	0.205
	MET	0.205	0.205
	FLIP	0.4	0.4

Cell line	Parameter	(−) 17AAG	(+) 17AAG
(B)			
HCC827 (3D)	EGFR	0.25	0.2
	MET	0.25	0.2
	Stress	0.6	0.6
	HSP60	0.4	0.4
	FLIP	0.7	0.6
	p53	0.4	0.4
	(Erb2/Erb3/c‐RET)	0.07	0.07
	Erb2/Erb3/c‐RET	0.1	0.07
	(EGF‐EGFR)	0.1935	0.1935
	(MET)	0.1935	0.1935
A549 (3D)	KRAS^c^	0.352	0.352
	Stress	0.5	0.5
	FLIP	0.7	0.33
	p53	0.0	0.0
	HSP60‐act	0.4	0.4
H441 (3D)	KRAS^c^	0.43	0.43
	EGFR	0.05	0.01
	Erb2/Erb3/c‐RET/FGFR3	0.04	0.03
	MET	0.05	0.02
	Stress	0.7	0.7
	p53‐act	0.65	0.65
	HIF1‐act	0.65	0.65
	HSP60‐act	0.05	0.05
	VEGFR2	0.35	0.33
	FLIP	0.75	0.75
	PTEN	0.41	0.41

Cell line	Parameter	(−) AICAR	(+) AICAR	(−) PX‐478	(+) PX‐478
(C)					
A549 (3D)	KRAS^c^	0.345	0.345		
	Stress	0.5	0.5		
	FLIP	0.6	0.2		
	p53	0.0	–		
	p53‐act	–	0.1		
	low glucose	0.3	0.3		
	HIF1‐act	0.8	0.8		
	mTOR‐act	0.65	0.65		
H441 (3D)	KRAS^c^			0.43	0.43
	EGFR			0.05	0.05
	Erb2/Erb3/c‐RET/FGFR3			0.04	0.04
	MET			0.05	0.05
	Stress			0.7	0.7
	p53‐act			0.65	0.65
	HIF1‐act			0.65	0.65
	HSP60‐act			0.05	0.05
	VEGFR2			0.35	0.05
	FLIP			0.75	–
	casp3‐act			–	0.75
	PTEN			0.41	0.41

^a^ Cell line‐specific receptor or pathway activity of proteins according to the experimentally determined differences in response behavior (apoptosis, proliferation, RTK, and western blot data); all other proteins were simulated with no specific activation. (−) Treatment activation at stage 0; (+) treatment activation at stage 1. ^b^ For the simulation of the AMPK activator AICAR in A549 and the HIF1A inhibitor PX‐478 in H441, we used the cell line‐specific activity from the untreated cells of the 17AAG treatment (Table 3C); all other proteins were simulated with no specific activation. (−) Treatment activation at stage 0; (+) treatment activation at stage 1. ^c^Constant activation, as there is a *KRAS* mutation in these cell lines.

To reveal – in a systemic approach – further relevant cell line‐specific drug targets in *KRAS*‐mutated conditions in the 3D system, we reconstructed two larger *in silico* networks for A549 and H441 cells. Therefore, we searched in Human Protein Reference Database (HPRD; Table S1) the interacting neighbors of the nine upon 17AAG treatment between A549 and H441 differentially regulated proteins (DRPs) (Table 1B, Fig. 6) and identified individual promising drug targets by mapping these to cell‐specific mutations in COSMIC (Catalogue Of Somatic Mutations In Cancer) generating thereby two cell‐specific networks for A549 and H441 cells. From networks analyses, we expanded the in this study created topology from Fig. 4B (marked with olive gray background in Fig. 7A,C) further with gain or loss of function mutations and other important factors by adding activated or inhibited nodes. In subsequent simulations we could predict optimal drug targets in a specific mutational background of *KRAS*‐mutated tumors. In Table 3C, input into topologies and subsequent simulations in Fig. 7 are given. Matching drugs were suggested by screening of our DrumPID (Drug‐minded Protein Interaction Database) (Kunz *et al*., 2016) screening tool for available target‐specific test substances.

**Figure 6:**
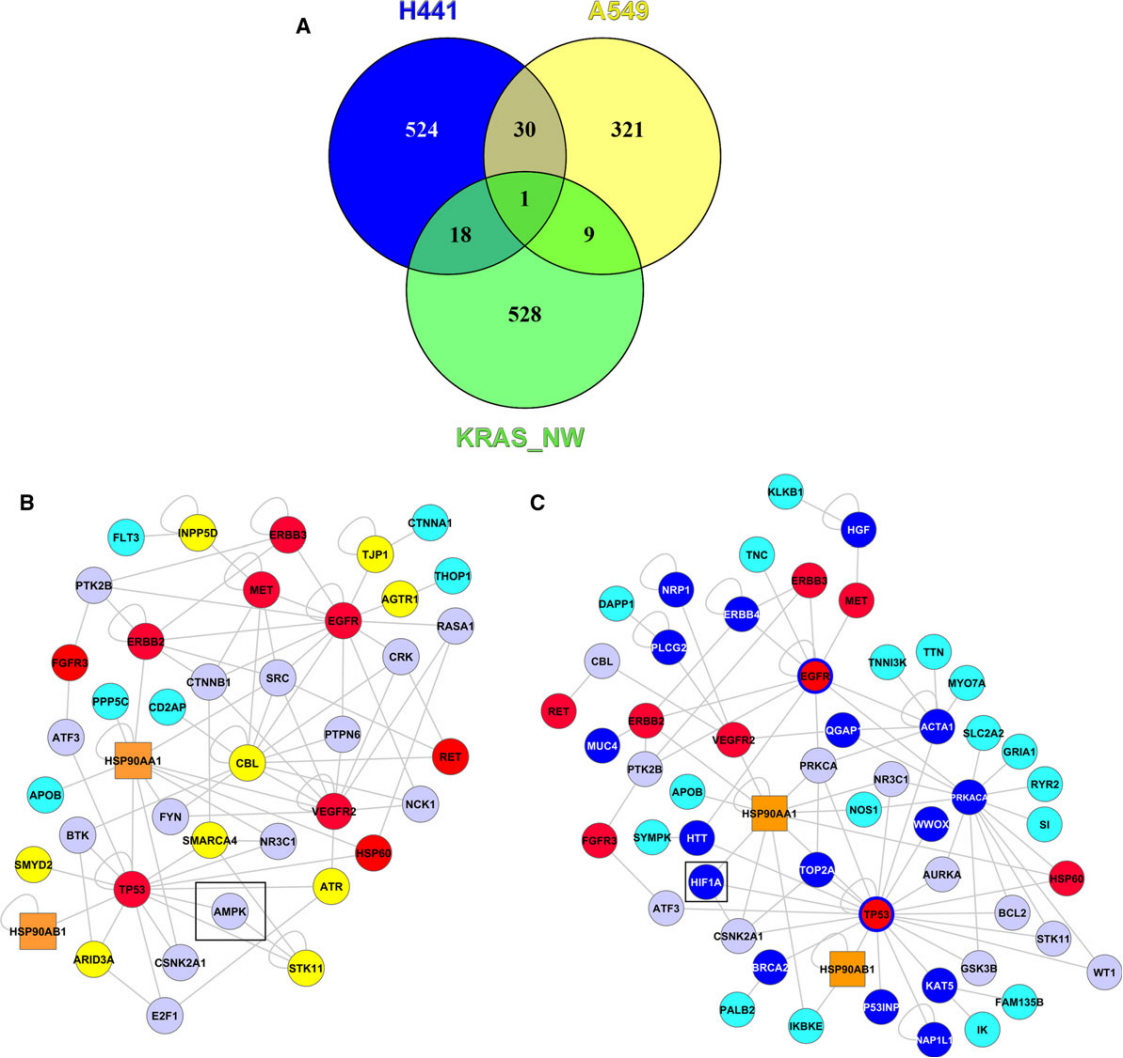
KRAS signature development and individual target predictions by generation of HPRD networks. We generated a network around KRAS, according to the experimentally validated DRPs between both KRAS‐mutated cell lines (H441, A549) in the 3D system (Table 1B; 17AAG treatment), and included their direct protein interaction partners using the genomewide HPRD. The resulting larger KRAS interaction network includes 556 proteins (= nodes) and 680 protein–protein interactions (= edges), around nine strongly DRPs (EGFR, ErbB2, ErbB3, MET, FGFR3, c‐Ret, VEGFR2, p53, and HSP60). (A) A Venn diagram compares cell line‐specific mutations. Mapping of cell line‐specific protein mutations (573 for H441 (blue) and 361 for A549 (yellow) from the COSMIC database) against the 556 proteins from the network around KRAS results in 18 H441‐specific mutations and in nine A549‐specific mutations which were included in each cell line‐specific *in silico* topology to yield the network. Details are given in the Supporting information, and key network differences are shown in B and C. (B) A549‐specific network: represents neighbor proteins that we could target if we consider the experimental data and directly interacting protein neighbors (from HPRD; functional clusters in Fig. S5A). As drug targets do not appear for these small modules from key signaling proteins, we considered experimental derived proteins (red) with all first‐degree neighbors, HSP90 (orange rectangle), and additionally direct neighbors to cell line‐specific mutations (in yellow, suspected ‘driver mutations’). Direct neighbor proteins are labeled in lavender, in cyan are neighbors from neighbors, which are also mutated. The black square (AMPK, interactor of p53 and LKB1) indicates a promising drug target (screening procedure given in Box S1). (C) H441‐specific network: shows neighbor proteins that we could target, if we consider the experimental data, HSP90 (orange rectangle) and directly interacting protein neighbors from HPRD (functional clusters in Fig. S5B). Directly interacting neighbors are shown (lavender, labeling binary interactions). As drug targets do not appear for these small modules from key signaling proteins, we considered all experimental determined nodes (red) with all first‐degree neighbors integrating cell line‐specific H441 mutations (in blue, suspected ‘driver mutations’; EGFR and p53 labeled in red with blue circles as they are array nodes and mutated). Protein interactors according to HPRD are labeled in lavender; in cyan are neighbors from driver mutations, also showing a mutation in H441. The square (HIF1A) indicates a promising drug target (screening procedure given in Box S1).

**Figure 7:**
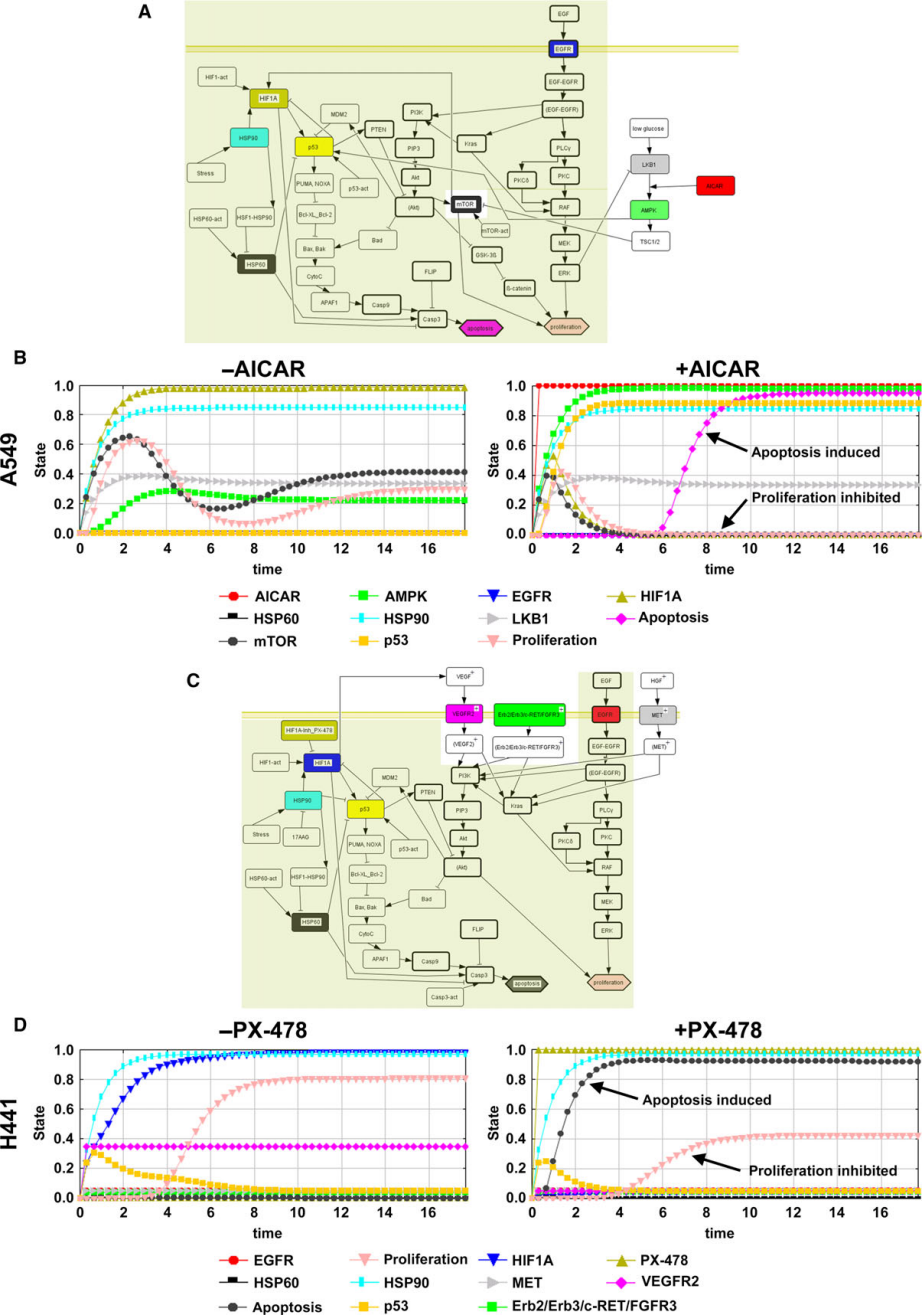
*In silico* topologies and simulations of AMPK and HIF1A treatment. Cell‐specific network extensions according to the experimental data (Table 2) are mapped into the shared topology (bold nodes from basic topology from Stratmann *et al*. (2014), olive shade for topology nodes from Fig. 4B). Furthermore, AMPK as a relevant target for A549 (network in (A)) and HIF1A as a target for H441 (topology in (C)) are included (nodes equivalent to the 17AAG treatment are deposited in olive, protein node colors are the same as in the simulation curves). Both protein targets were integrated with their direct interacting protein neighbors in the cell‐specific networks to mirror *in silico* individual therapy. In (B) and (D), the cell‐specific topologies are next simulated dynamically, and selected trajectories of protein node activities were plotted, showing the effects of the potential drug candidate AICAR as an AMPK activator for A549 (B), and the HIF1A inhibitor PX‐478 for H441 in (D) to illustrate the *in silico* screen of different drugs in the two cell line‐specific topologies. (B) Simulation of AMPK activation in A549 cells (right, red curve at stage 1) results in higher apoptosis (pink curve) and reduced proliferation (salmon curve), as compared to untreated cells (left, red curve at 0.0, no activation). (D) The *in silico* simulation of the HIF1A inhibition for H441 (right, olive curve at full activation) shows higher apoptosis (black curve) and reduced proliferation (salmon curve), as compared to untreated cells (left, olive curve at 0.0, no activation).

### 2.2 Tissue‐engineered lung tumor models resemble tumor specimens

Firstly, we looked at molecular markers for tumors and tissue differentiation and their variances. We observed homogenous E‐cadherin/β‐catenin localization in fluorescence staining of 3D models as well as tumor specimens, whereas 2D models showed high variation ranging from strongly stained to completely negative tumor cells (Fig. 1A,B). By comparison to 2D models, we demonstrated with Ki67‐staining the reduction in proliferation rate in 3D models to levels that correlate to lung adenocarcinoma samples (Fig. 1C).

### 2.3 Enhanced biomarker‐dependent drug response to EGFR inhibition in the 3D model

As a test for clinically applied biomarker‐guided anti‐EGFR therapy, we compared A549 and H441 (EGFR wild‐type) with HCC827 cells (activating *EGFR* mutation). Responses upon 3 days of Gef treatment were biomarker‐dependent, as represented by hematoxylin and eosin staining (HE staining) in 3D models (Fig. 2A), proliferation reduction (Fig. 2B), and apoptosis induction (Fig. 2C). Although the proliferation in 3D conditions was reduced to *in vivo* like rates (Stratmann *et al*., 2014), treatment with Gef decreased the proliferation further by about 80%, as also observed in 2D. However, biomarker‐related apoptosis induction upon Gef treatment in HCC827 was significantly enhanced in 3D conditions (about 3.5 to 6‐fold increase), compared to 2D conditions (about 2.5‐fold increase), which suggests better specificity for the 3D system. Signaling analyses by receptor tyrosine kinase (RTK) and phospho‐kinase (PK) array experiments are provided for the 2D and 3D systems (Fig. S1, Table 1A).

Complementing these biomarker‐dependent drug responses to Gef, we set up an *in silico* network of key pathways for the proliferative and apoptotic response, to model the observed *in vitro* responses of each of the three cell lines. An *in silico* topology was previously developed for HCC827 and A549 (Stratmann *et al*., 2014). This was extended by those cell line‐specific proteins and pathways (Fig. S2A; previous network proteins are in bold and with olive background) which showed signaling changes upon the Gef treatment in the experiments (Table 2). For the newly investigated H441 cell line, we used the A549 *in silico* topology as basis. Specifically, we integrated the MET signal transduction cascade for HCC827 and H441. We then applied the squad software to simulate the Gef treatment responses for all cell lines, using initial node stimulations based on the mutational background and the experimental results on protein phosphorylation (prestimulation in Table 3A; method in the Supporting information). Our simulation of HCC827 with Gef treatment (Fig. 2D) compared to untreated cells demonstrates, as for the *in vitro* results, reduced proliferation and higher apoptosis over time. Results of the simulations for A549 and H441 are represented in Fig. S2B.

### 2.4 Chemoresistance against HSP90 inhibition in 3D models align to clinical observations

2D models and animal experiments predict HSP90 inhibitor efficiency in *KRAS*‐mutated tumors (Acquaviva *et al*., 2012; Sos *et al*., 2009). As known from 2D *in vitro* screens, HCC827, A549, and H441 exhibit different sensitivities to the HSP90 inhibitor 17AAG (Ciocca and Calderwood, 2005; Sos *et al*., 2009).

We observed that about 50% of the H441 cells died from 0.25 μm 17AAG, which decreased the viability of A549 to 5% and of HCC827 to 35%, as shown by the cell viability assay CellTiter‐Glo^®^ in 2D (Fig. 3A). However, the failure of HSP90 inhibitor treatment in a clinical setting of *KRAS*‐mutated tumors was reflected in 3D tissue cultures. From HE staining of 3D tumor models, after three days of 0.25 μm 17AAG treatment only slight effects were visible in A549 and HCC827, whereas H441 cells were completely unresponsive (Fig. 3B). Proliferation analysis of the 2D systems predicts *KRAS*‐mutated cells to be more responsive to 17AAG than *KRAS* wild‐type HCC827 cells. This is contrasted by only weak changes between both cell types in 3D tissue culture (Fig. 3C). A strong apoptotic response upon 17AAG is only observed in *KRAS*‐mutated A549 cells in 2D (4 to 6‐fold) but not in 3D models (1 to 2‐fold) (Fig. 3D).

All phosphorylation data from arrays and western blot experiments are summarized and compared in 2D and 3D models in Table 1 for Gef (A) and 17AAG (B) treatment. Protein nodes for *in silico* topology which were applied later are given in Table 2.

### 2.5 Differences in signaling between 2D and 3D conditions upon HSP90 inhibitor treatment as a basis for *in silico* analyses

Signaling responses upon application of the HSP90 inhibitor 17AAG were analyzed by comparing 2D and 3D conditions. Protein activation as observed by RTK arrays was confirmed by western blot (Fig. 4A) and quantified (Fig. S3B). Data indicated an inhibition of the EGFR and of MET in HCC827 and in H441 in 2D as well as 3D conditions. In western blot analysis, inhibition of MET was weaker in 3D than in 2D cultures in both cell lines. Interestingly, p53 (S46) was activated in HCC827 with 17AAG treatment in 2D, but stayed constant in 3D conditions. *Vice versa*, in H441 p53 was activated only in 3D conditions and remained unchanged in the 2D culture. Furthermore, HSP60 was clearly upregulated only in A549 cells under 3D conditions upon 17AAG application. Regulated proteins identified in 3D conditions upon 17AAG treatment include EGFR, ErbB2, ErbB3, MET, c‐Ret, VEGFR2, FGFR3, p53, and HSP60 (Table 1B, Figs 4A, and S3A). Similar to the Gef treatment, we extended our *in silico* network and topology adding these experimentally measured cell‐specific proteins (Fig. 4B). Particularly, we included for mirroring 17AAG treatment effects – next to the MET protein – ErbB2, ErbB3, and c‐RET cascade in HCC827 and H441, and also in all three cell line‐specific *in silico* topologies p53, HSP60, HIF1A, and HSP90, as part of the 17AAG treatment cascade (Table 2). For H441, we included further VEGFR2 and FGFR3, as they were downregulated in the arrays in the 3D model upon treatment with 17AAG, in contrast to the other two cell lines (Fig. S3A, Table 1B). We show only responses for key proteins of all three cells, but we simulated the complete network responses looking at all proteins of the topology. Important aspects of the 3D tissue model upon 17AAG application (red curve at 1) are reflected by *in silico* simulations (Fig. 5): (a) In HCC827 (top), cell proliferation is unchanged and apoptosis is slightly induced compared to the untreated control, (b) in A549 (middle), proliferation is regarded as unchanged and apoptosis is only slightly induced in 3D conditions, and (c) in H441 (bottom), proliferation is unchanged and apoptosis is not induced. Notably, HSP60 is exclusively induced in A549, whereas p53 is upregulated only in H441. Moreover, based on the *in silico* topology connectivity, in our *in silico* simulation we found that beside p53 HIF1A is also upregulated in H441.

For comparison, the *in silico* simulations can also be modified to appropriately reflect results of 2D culture. To illustrate this, we focused on the A549 and H441 cell lines and applied the same topology as for 3D, but adjusted activation levels for Gef and 17AAG treatment according to the 2D *in vitro* conditions (Fig. 4B and S2A; Table 1; Box S2): Essentially, we elevated the value of Raf to simulate higher basic proliferation in 2D, and furthermore, we changed FLIP for the higher apoptotic response in 2D upon 17AAG as this is reported to be important for higher apoptotic resistance when cells grow on collagen (Philippi *et al*., 2009). Whereas Gef treatment simulation resulted in both *KRAS*‐mutated cells in no change of proliferation and apoptosis over time (Fig. S6), HSP90 inhibition simulation of 2D conditions revealed in contrast to 3D a lower proliferation and an induced apoptosis over time in A549 (Fig. S7).

However, the established tool allows us now to test and screen *in silico* in a systems perspective for tailored therapies according to the cell line‐specific mutational profile and tumor drivers, as detailed in the following section.

### 2.6 Generation of *in silico* protein–protein interaction networks for cell‐specific drug target predictions in *KRAS*‐mutated cells

Next, as we observed signaling differences between the *KRAS*‐mutated A549 and H441 cell lines, we sought to identify a *KRAS* complementing signature of further potential biomarkers and resultant drug targets for each cell line. For this purpose, we combined experimental data and the cell line‐specific mutational backgrounds with integrated systems biology analysis (Kunz *et al*., 2017; Naseem *et al*., 2014), considering direct interacting proteins and available drugs to modulate this extended network.

We generated a network around KRAS by considering the DRPs of both *KRAS*‐mutated cell lines (A549 and H441) upon 17AAG treatment (Table 1B) in the 3D system and included their direct interacting proteins according to the genomewide HPRD. The resulting KRAS interaction network includes 556 proteins (= nodes) and 680 protein–protein interactions (= edges) around the nine experimentally DRPs (EGFR, ErbB2, ErbB3, MET, FGFR3, c‐Ret, VEGFR2, p53, and HSP60; Fig. S4A). Comparing all cell line‐specific mutations known from the genomewide COSMIC database (573 for H441, blue circle, and 361 for A549, yellow circle; Table S2; Fig. 6A) with this KRAS interaction network, we could match 18 H441‐specific mutations and nine A549‐specific mutations, as parts of our KRAS interaction network (Table S3). The two reconstructed cell‐specific KRAS interaction networks for A549 and H441 included these specific mutations, HSP90 as a target of 17AAG, and their direct interaction partners from HPRD (Fig. S4B–D), which were then analyzed for functional clusters. In the A549‐specific network (322 proteins and 371 protein‐protein interactions, Fig. S4B; extended network with 795 nodes and 1034 interactions in Fig. S4C), we found two functional protein clusters with a strong network effect (so‐called hubs) around proteins VEGFR2, MET (experimental measurements) and CBL (mutated), and p53 (experimental) and ARID3A (mutated; Fig. S5A). Similarly, for the H441‐specific network (903 proteins and 1119 protein–protein interactions; Fig. S4D), we found two clusters around the proteins PRKACA (mutated) and p53 (experimental and mutated) as well as HSP90AA1, ACTA, and HIF1A (mutated; Fig. S5B). We compared potential targets in the two cell line‐specific KRAS networks, in terms of their distance and usefulness to modulate cell‐specific signaling cascades. This yielded a highly connected network between interesting tumor drivers (Fig. 6B for A549, driver mutations in yellow; Fig. 6C for H441, driver mutations in blue), and cell‐specific biomarker signatures (Table 4, Box S1). Other cell‐specific mutations close to the central cascade are indicated by cyan (neighbors of neighbors), and unmutated interactors in lavender circles for A549 and H441, respectively. Regarding ranking of drug targets for potential clinical application, we considered proteins and connections and assigned the priority to direct neighbors, if they could be targeted easily by existing medical drugs, for example, AMPK for A549 and HIF1A for H441. All targets are ranked in Box S1. This drug‐search strategy was made possible by applying our DrumPID (Kunz *et al*., 2016).

**Table 4:**
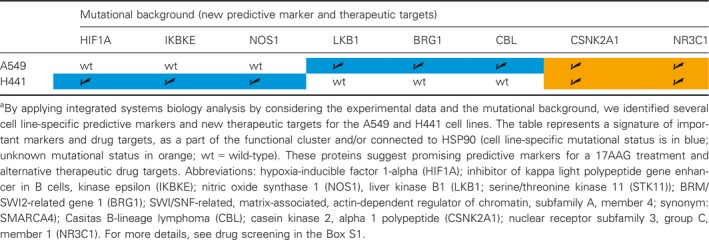
Overview of potential predictive markers and new therapeutic targets for the *KRAS*‐mutated cell lines^a^

### 2.7 AMPK and HIF1A targeting in cell‐specific *in silico* simulations for A549 and H441 cells

Subsequently, we investigated *in silico* the potential therapeutic effect of AMPK as a relevant target for A549 and HIF1A as a target for H441. For this, we integrated the LKB1 cascade for A549 into the cell‐specific *in silico* topology that simulated the 17AAG therapy (Fig. 7A), and further considered the connectivity of HIF1A in H441 (Fig. 7C). Proteins in the network that correspond to the basic topology of Stratmann *et al*. (2014) are bold rimmed and proteins that match to the topology from Fig. 4B have an olive background (Fig. 7A,C). From our drug screening (Box S1), we identified 5‐aminoimidazole‐4‐carboxamide ribonucleotide (AICAR) in A549 as the potential activator of AMPK that is directly modulated by its interactor LKB1, which is specifically mutated in A549 (synonym: STK11; see DrumPID pathway ko04152). Similarly, we found PX‐478 as a selective HIF1A inhibitor for the H441 cell line. Based on this, we simulated their potential therapeutic effect by applying the SQUAD algorithm (Fig. 7B,D; prestimulations in Table 3C). For *in silico* simulations of AMPK activation and inhibition of HIF1A, we found induced apoptosis and reduced proliferation in each cell line over time as a desirable drug effect.

## 3 Discussion

### 3.1 Motivation

Personalized treatment strategies have to cope with highly redundant tumor pathways resulting in resistance, whereas combination therapies often show severe toxic side effects (Tannock and Hickman, 2016). Therefore, it is necessary to reconsider the design of clinical studies with targeted anticancer approaches. It is critical to understand the underlying dependencies in signaling networks, and to provide tools for exploiting now frequently available sequencing data from patients.

Moreover, in the field of oncology, the success rate of preclinical testing is at under 5%, generating enormous financial costs (Bhattacharjee, 2012). Even though animal models predict toxicity quite convincingly, they tend to fail in efficacy testing (Greaves *et al*., 2004; Kubinyi, 2003). In particular, for signaling analyses, mice are not adequate models due to inappropriate ligands to some centrally connected human receptors, such as MET (Francone *et al*., 2007). Next to ethical concerns, these aspects underline the urgent need to develop novel human tumor test systems.

Here, we introduce a new concept of *in vitro* tissue tumor models and *in silico* analyses to design and test individual biomarker profiles and intervention strategies. This prepares the floor for patient‐tailored clinical studies, required for personalized cancer medicine (Tannock and Hickman, 2016). As our main aim is to develop a powerful tool that can be implemented into the clinic by analyzing the patient's sequence data, we investigate as proof of concept in this work three individual lung cancer cell lines with known genome sequence information. However, here we show only exemplary data and not a large validation series. We are aware that exact quantitative estimates require a higher number of experiments and more cell lines with similar driver mutations. Exploring its clinical implementation, currently our *in silico* tool advises on a case‐by‐case basis the molecular tumor board in our comprehensive cancer center. In particular it supports to identify alternative protein targets when resistance to treatment occurs.

Our human 3D tumor model generated by tissue engineering technologies should reduce preclinical failure, as it reflects tumor characteristics better and shows higher predictive accuracy than conventional 2D cultures: it retains the tissue architecture, extracellular matrix components and structures of the basement membrane as unique features for cellular interactions. These are important modifiers of cellular responses (Linke *et al*., 2007; Philippi *et al*., 2009; Schanz *et al*., 2010). In detail, we observed (a) more homogenous staining of E‐cadherin/β‐catenin and lower proliferation rates according to tumor specimens, (b) a biomarker‐dependent apoptosis induction and proliferation reduction by the EGFR inhibitor Gef, and (c) in contrast to other preclinical findings, a reduced response upon HSP90 inhibitor treatment in *KRAS*‐mutated tumor cells, which matches observations from clinical studies. Thus, we believe that our *in vitro* model resolves interpathway dependencies more reliably than 2D or animal models.

Individual KRAS *in silico* networks were established by integrating relevant proteins from *in vitro* experiments and their interaction partners from HPRD. Our simulations start from a general *in silico* network for lung cancer, which is refined here to reveal the most relevant protein clusters. By matching cell line‐specific mutations from the COSMIC database, we derived individual drug targets and by screening our custom‐made, protein–drug interaction database DrumPID appropriate drugs (Kunz *et al*., 2016).

### 3.2 HSP90 inhibition in *KRAS*‐mutated tumors and correlation of our 3D tissue models and other preclinical models to clinical findings

In a previous study of a lung cancer model, we were able to demonstrate a stronger apoptosis induction in the 3D model by Gef, compared to conventional 2D culture (Stratmann *et al*., 2014). After setting up standard operating procedures (Göttlich *et al*., 2016), we could predict the clinical failure of HSP90 inhibitor treatment in the context of *KRAS* mutation, in contrast to other *in vitro* and *in vivo* models (Acquaviva *et al*., 2012; Sos *et al*., 2009). Heat shock proteins have gained attention in recent years as therapeutic tools, as they are involved in tumor cell proliferation, invasion, and cell death. Their high expression was observed in several cancer entities in clinical settings (Ciocca and Calderwood, 2005). Specifically, HSP90 belongs to a family of chaperons important for the function of relevant oncogenic drivers in lung adenocarcinomas. From a genomewide screening of 84 cell lines, *KRAS* mutation was identified to confer sensitivity to HSP90 inhibition that could also be verified in murine models (Sos *et al*., 2009). In this screening, the geldanamycin derivatives 17AAG, and 17‐dimethylaminoethylamino‐17‐demethoxygeldanamycin in mice experiments were applied for HSP90 inhibition. However, geldanamycin and its derivates turned out to have safety and pharmacological limitations (Jhaveri and Modi, 2015). Another *in vitro* study showed the effectiveness of HSP90 inhibition in several *KRAS*‐mutated non‐small cell lung cancer (NSCLC) lines by ganetespib – a non‐geldanamycin analog with less toxic side effects (Acquaviva *et al*., 2012). However, single agent HSP90 inhibition by ganetespib failed in NSCLC patients with *KRAS*‐mutated tumors. Combination therapy trials with docetaxel (GALAXY 1 and 2) led to better outcomes in patients with adenocarcinomas, than docetaxel single agent therapy, but not in the subgroup of *KRAS*‐mutated tumors (Bhattacharya *et al*., 2015). Recently, ALK, ROS1, and RET kinase gene rearrangements have been suggested to predict efficacy by targeting HSP90 (Rothschild, 2015; Sang *et al*., 2013; Socinski *et al*., 2013).

### 3.3 *In silico* simulations of cell responses and development of a predictive *KRAS* signature

In this study, *in silico* analyses for *KRAS* signature development are executed in three steps:

1. Set up of cell‐specific *in silico* topology with logical Boolean connectivity (software tool celldesigner; http://www.celldesigner.org; Funahashi *et al*., 2008)
2. Cell‐specific dynamic *in silico* simulations of tumor cell responses (software tool squad)
3. For systematic drug‐target identification we generate larger cell‐specific protein–protein interaction networks considering neighbors of the central cascades (using data from HPRD) and cell line‐specific mutations (using data from COSMIC). For drug suggestions we apply the database tool DrumPID.

In detail, we explain here the three above mentioned distinct types of *in silico* analyses:

1. With the term ‘*in silico* topology’, we considered a previously published knowledge‐based network which focuses mainly on kinase cascades (Stratmann *et al*., 2014) and integrated here cell‐specific differences as additional nodes (proteins) derived from experimental data to specifically mirror the effects of Gef treatment and HSP90 inhibition. For this, we measured by phospho‐arrays and western blot signaling changes as drug responses as well as differences in proliferation and apoptosis in the different cell lines in 2D and 3D conditions. Missing parts of the cascade or modulatory crosstalk are filled in according to expert knowledge and public databases. These represent only the key parts of the signaling cascades. We used the tool ‘CellDesigner’ to set up the *in silico* topologies and to bring them into a machine‐readable format as done before for other cell types (Schlatter *et al*., 2012).
2. ‘*in silico* simulations’ with the SQUAD tool predict the systemic response of a tumor cell upon a specific treatment which depends on the tumor cell topology and the activation/inactivation of its integrated nodes. As input the activation level of certain nodes can be set between zero as an inhibitory effect (inactivation) and one as an activating effect (activation). Furthermore, mutations can be integrated that stay independent from upstream signaling events at a certain value in case of gain or loss of function mutations. Also differences in 2D and 3D conditions can be simulated by adjusting the nodes’ values to experimentally measured levels, that is, phosphorylation determined by western blot. Some of the nodes summarize also global cellular responses, for example, ‘stress’. Importantly, also the drug responses proliferation and apoptosis are integrated in the topology as nodes. Values of the other nodes must be adjusted until the level of proliferation and apoptosis comply with the *in vitro* observations. Traditionally, differential equations for detailed kinetic modeling look at biological responses (Di Cara *et al*., 2007; Dwivedi *et al*., 2015; Robubi *et al*., 2005). However, this requires then detailed kinetic information on individual kinases. This is not necessary in our approach, as the squad modeling software interpolates automatically exponential functions between our protein network nodes fitting signal transmission and logical connectivity (Di Cara *et al*., 2007). We previously applied this combination to study cancer (Göttlich *et al*., 2016; Stratmann *et al*., 2014), infection biology (Audretsch *et al*., 2013; Naseem *et al*., 2012), and different tissues (Brietz *et al*., 2016; Czakai *et al*., 2017; Philippi *et al*., 2009).
3. For drug targeting, we looked systematically at larger protein–protein interaction networks; in particular, we collected all neighbors of upon 17AAG treatment between DRPs of both *KRAS*‐mutated cell lines. To this network, we matched cell‐specific mutations from the COSMIC database. These larger networks we term here ‘*in silico* networks’. The cell‐specific networks were then scrutinized to identify most promising treatment targets considering their relation to highly connected proteins in the network that are called ‘hubs’. A robust drug prediction algorithm collates information from several large‐scale databanks including chemical information according to Simplified Molecular Input Line Entry Specification (SMILES) notation and basic drug pharmacokinetics ADME (absorption, distribution, metabolism, excretion) rules (DrumPID, Kunz *et al*., 2016). We used our reconstructed cell‐specific networks (Fig. 6B,C) and screened which drugs according to DrumPID (Kunz *et al*., 2016) influence apoptosis and proliferation in a cell‐specific manner. Targets are ranked by the effect strength, closeness to central cascades and druggability (Box S1). Subsequently, we simulated the potential therapeutic effect on apoptosis and proliferation focusing on AICAR (AMPK activator) and PX‐478 (HIF1A inhibitor) as top candidates and integrated their specific connectivity to the central cascades in the *in silico* topology. However, other drug target candidates can also be simulated, but for each simulation the individual targets and side targets of the drug has to be considered. Further testing of predictions is required to confirm suggested targets regarding clinical relevance. So far, neither HCC827, nor A549, nor H441 lung cancer cell lines have been analyzed by such a comprehensive *in silico* approach.

### 3.4 Experimentally measured differences between the 2D and 3D system and *in silico* analyses

Besides a higher chemoresistance in the case of HSP90 inhibitor treatment, we observed in 3D lower reduction of MET upon 17AAG treatment and an inverse regulation of p53, when compared to 2D conditions. Next to semiquantitatively evaluated western blot experiments, we present data from two phospho‐array screens (RTK, PK) as a starting point for further analyses (Figs S1 and S3A).

Importantly, in our 3D experiments we observed in contrast to 2D conditions upon 17AAG treatment an upregulation of HSP60 exclusively in A549, and an activation of p53 only in H441, which we were able to achieve also in our *in silico* simulations. However, the literature reports HSP60 inhibition by HSP90 and p53 inactivation by HSP60 (Ghosh *et al*., 2008), which would explain our experimental observations in A549 and H441. The reason why HSP60 upregulation and a lack of p53 expression in A549 has a small effect on apoptosis in this setting, could be due to reduced HIF1A activation upon 17AAG treatment, as predicted by our *in silico* simulation. This reduced activation could stem from the inhibition of HSP90. HIF1A is not completely silenced in the simulation, due to its connection to LKB1 via mTOR in the *in silico* topology, as according to the COSMIC database, in A549 LKB1 carries a loss of function mutation. Furthermore, our *in silico* topology illustrates that this LKB1 mutation should lead to reduced AMPK activation and, thereby, also reflects the nonproliferative effect of 17AAG treatment via the mTOR signaling pathway. On the other hand, apoptosis induction is also blocked in H441. Induction of p53 upon inhibition of HSP90 should have no apoptotic effect due to the loss of function mutation of p53 identified in the COSMIC database. Furthermore, in our simulation we can see that HIF1A is still activated in H441 following HSP90 inhibition. This could be due to a mutation inside this gene that leads to an inhibitory effect on apoptosis and favors proliferation. Box S2 shows that we can use the same topology to simulate 2D results *in silico*, but differences in protein activation have to be taken into account to appropriately simulate the stronger apoptotic as well as proliferative responses upon 17AAG treatment observed in 2D cultures. As we could correctly simulate the observed responses upon Gef and 17AAG treatment for 2D and 3D conditions we could support that nodes in our topology are so far connected correctly.

The *in silico* screen can reveal new dependencies, as high quality databases consider cancer‐subtype‐specific mutations and their interacting proteins along with all available drugs to directly attack the mutated protein or one of its neighbor.

AICAR and PX‐478 are given as attractive examples (top ranked; see Supporting information) and their therapeutic effect on apoptosis and proliferation is simulated. However, other drugs can also be used by integrating other drug target candidates by considering its individual targets and side targets.

Subsequently, the targets and drugs can be integrated in the *in silico* topology by considering its specific connectivity to the central cascades and further *in silico* simulated with SQUAD.

### 3.5 Exemplified target and drug candidate prediction for A549 cells

From the newly established KRAS network, based on proteins that exhibit changes in signaling between A549 and H441 in 3D conditions and cell‐specific mutations, the for A549 unique mutation LKB1 stands out. Our drug–protein interaction database DrumPID (Kunz *et al*., 2016) identifies drugs that modulate the query protein directly, or one of its directly interacting neighbor proteins. This database tool identifies AMPK in our analyses as a potential drug target in A549 cells which can be modulated by the drug AICAR. AMPK protein is a direct interaction partner of LKB1 (Fig. 6B) (Fay *et al*., 2009; Rattan *et al*., 2005; Tang *et al*., 2011). AMPK activation using the drug AICAR, an analog of AMP, leads to tumor growth arrest in our *in silico* simulation for A549 cells (Fig. 7A,B; nodes from first topology are olive‐shaded in 7A). Moreover, AICAR shows promising results in the clinical phase 1/2 for chronic lymphatic leukemia (Van Den Neste *et al*., 2013). In addition, the approved anticancer agent pemetrexed is known to indirectly activate AMPK by the accumulation of ZMP in LKB1‐null lung cancer (Rothbart *et al*., 2010).

### 3.6 Exemplified target and drug candidate prediction for H441 cells

Regarding H441 cells, we also screened the H441 protein interaction network around the KRAS signature for potential drugs targeting either the protein or its direct neighbor. HIF1A was the highest‐ranked target (Box S1), as it is altered in H441 cells according to COSMIC data and is involved in a signaling loop (Greijer and van der Wall, 2004) (Fig. 6C). Inhibition of HIF1A using PX‐478 shows an antitumor effect in our *in silico* simulations (Fig. 7C,D; nodes from first topology are olive‐shaded in 7C). Studies demonstrated that inhibition of HIF1A shows promising therapeutic effects in human xenograft models (Welsh *et al*., 2004).

### 3.7 Application of the combined *in vitro*/*in silico* tool

For clinical application, patient tumors have to be sequenced first, or at least tested by PCR or microarrays, to confirm that the driver mutation profile matches those in our cell lines. Notably, primary tumor cell culture is still challenging and has to be optimized for its utilization in routine personalized approaches.

## 4 Conclusion

Predictive gene signatures were identified in a combined, tissue‐engineered, 3D lung tumor model with improved clinical correlation and a Boolean *in silico* approach that integrated measured cell‐specific differences in drug responses. We established cell line‐specific networks that depend on individual mutation patterns. This enabled better understanding of the interdependencies between single signaling cascades to prevent treatment resistance. Exemplified by the *KRAS*‐mutated cell lines A549 and H441, we demonstrated how our analysis tool could lead to individual signature development, based on *in vitro*/*in silico* investigations on signaling, interaction partners from the HPRD, and sequence data from COSMIC. The limited number of direct interference points with the proliferative and/or apoptosis signaling cascade suggests and ranks best cell line‐specific targets, implying future therapies according to NGS data, tailored to the individual cancer mutation profile. Translated into clinical application, our lung cancer cell line‐specific examples suggest for patient stratification to determine not only the *KRAS* mutation status, but also to test for LKB1, p53, and HIF1A. Such cancer‐specific prescreening could distinguish among individual mutational subgroups to improve patient stratification and the design of clinical studies.

## 5 Materials and methods

### 5.1 Cell culture

HCC827 and A549 cell lines were purchased from DSMZ (Braunschweig, Germany), H441 from ATCC (LGC Standards GmbH – Germany Office, Wesel, Germany). A549 and H441 cells were cultured in RPMI + 10% FBS, HCC827 cells in RPMI + 20% FBS. Cells were monitored for pathogen infections at regular intervals. For a 2D culture, cells were either grown on glass coverslips in well plates until they had reached a confluency of about 70% or were cultured for 5 days in 12‐well plates or 6‐cm petri dishes. For a 3D culture, 1 × 10^5^ tumor cells were grown for 14 days on the SISmuc (see Section 5.3) that was fixed between two metal rings, as described in the literature (Göttlich *et al*., 2016; Moll *et al*., 2013; Stratmann *et al*., 2014). Both 2D and 3D cultures were performed under standard conditions (37 °C, 5% CO_2_).

### 5.2 Treatment with Gef and 17AAG

After 1 day in a 2D and 11 days in a 3D culture, cells were treated with either 1 μm Gef (Iressa™, AstraZeneca, Wedel, Germany; Selleckchem) or 0.01, 0.05, 0.1, 0.25, 0.5 or 1 μm 17AAG (17‐*N*‐allylamino‐17‐demethoxygeldanamycin, Tanespimycin; Selleckchem) for 72 h, with a medium change after the first 48 h of treatment.

### 5.3 Porcine material

The SISmuc consisting of porcine small intestine submucosa (SIS) and mucosa (muc) was used as a scaffold for all 3D culture experiments. It was prepared from the BioVaSc^®^ as described in the literature (Linke *et al*., 2007; Schanz *et al*., 2010). All explantations were in compliance with the German Animal Protection Laws (§4(3), supervised by the institute's animal protection officer, all animals received proper care according to the National Institute of Health standards (NIH publication no. 85e23, revised 1996)), and as approved by the institutional animal protection board.

### 5.4 Human material

Human lung tumor tissue was provided by the Department of Thoracic Surgery of the University Hospital of Wuerzburg (local ethics approval: 182/10, 25.11.2015).

### 5.5 Histology and immunofluorescence

Cells cultured on glass slides in 2D were fixed in 4% paraformaldehyde for 10 min, cells in a 3D culture for 2 h, and the human lung tumor tissue overnight at 4 °C. The SISmuc samples, as well as the tumor tissue, were embedded in paraffin and sectioned at 3 μm thickness for hematoxylin–eosin (HE) and immunofluorescence staining. The primary antibodies E‐cadherin (#610181; BD Transduction Laboratories, Heidelberg, Germany), β‐catenin (#ab32572; Abcam, Cambridge, UK), and Ki67 (#ab16667; Abcam) were diluted 1 : 100 and incubated overnight at 4 °C. Secondary antibodies conjugated with fluorescent dyes Alexa 555 or 647 were diluted 1 : 400 and incubated for 1 h at room temperature. Nuclei were counterstained by DAPI dissolved in a Mowiol embedding solution. Pictures were taken with a digital microscope (BZ‐9000; Keyence Deutschland GmbH, Neu‐Isenburg, Germany).

### 5.6 Cell proliferation

To determine the proliferation rate, cells cultured in 2D and 3D were stained against Ki67. Ten nonoverlapping images of 3D sections and five nonoverlapping images of 2D cultures were taken. Quantification of the proliferation rate was performed as described in the literature (Göttlich *et al*., 2016).

### 5.7 M30‐elisa

Apoptosis was determined from supernatants taken from untreated and treated tumor models during the last 4 days of the culture. M30 CytoDeath™ ELISA (Peviva) was performed according to the manufacturer's instructions. All samples were measured in duplicates.

### 5.8 Western blot and phospho‐RTK and PK arrays

Cells were lysed in modified RIPA buffer (137 mm NaCl, 50 mm NaF, 20 mm Tris/HCl pH 8.0, 2 mm EDTA, 10% (v/v) glycerol, 1% (v/v) NP‐40, 0.5% (w/v) DCA, 0.1% (w/v) SDS, 1 mm Na_3_VO_4_, and 1× protease inhibitor cocktail (Sigma‐Aldrich, Darmstadt, Germany)), or in the provided lysis buffers of the respective array kit. For western blot analysis, protein samples (27 μg per lane) were separated electrophoretically in a 10% SDS/gel and blotted on a 0.2‐μm nitrocellulose membrane (Whatman, Fisher Scientific GmbH, Schwerte, Germany). The primary antibodies pEGFR (#ab32430; Abcam), pMet (#3077; Cell Signaling Technology, Frankfurt a. Main, Germany), phospho‐p53 (S46) (#2521; Cell Signaling Technology), HSP60 (#ab46798; Abcam), and β‐actin (#3700; Cell Signaling Technology) were incubated in NFDM or a 1% BSA overnight at 4 °C. Secondary anti‐mouse or anti‐rabbit IgG antibodies conjugated to horseradish peroxidase (#JAC‐111035144 or #JAC‐115035146; Jackson ImmunoResearch, Cambridgeshire, UK) were incubated for 1 h at room temperature. Bands were visualized using the Pierce ECL Western Blotting kit (Thermo Scientific, Breda, Netherlands). Phospho‐RTK and PK arrays were performed according to the manufacturer's instructions. Western blot and array membranes were imaged at the imaging station FluorChem Q (Biozym Scientific, Hessisch Oldendorf, Germany). Gray values were determined with the related image acquisition and analysis software alphaview (version 3.2.2.0; Proteinsimple, San Jose, CA, USA).

### 5.9 Statistical analysis of the experimental data

The nonparametric Kruskal–Wallis test and post hoc Wilcoxon rank‐sum test were used for statistical analysis of proliferation and apoptosis results. *P* < 0.05 was considered as significant. Statistical analysis was carried out with the open‐source software r (The Comprehensive R Archive Network).

### 5.10 Bioinformatics analysis

#### 5.10.1 Network analysis

Bioinformatics analyses combined cell culture array and western blot data for the Gef and 17AAG treatment in the 3D system with information from databases to build up an individual network for each cell line. We extended the original *in silico* topology (Stratmann *et al*., 2014) by integrating proteins listed in Table 2.

#### 5.10.2 Dynamic simulation

For the 3D *in vitro* system, we simulated the Gef and 17AAG treatment using the squad software (Di Cara *et al*., 2007), by taking the pathway activity differences into account (Table 2) while running the simulation (prestimulations in Table 3). For the 2D system we focused on the A549 and H441 cell lines (prestimulations in Box S2). SQUAD represents the network topology (activation, inhibition) using logical Boolean operator (AND, OR, NOT) and interpolates them by applying mathematical e‐functions. The resulting network effects are visualized in a graph as changes of state over an arbitrary time, allowing *in silico* simulations of different network scenarios. Simulation protocols were written using the SQUAD function ‘perturbator’ (prestimulation option in the simulation menu of the software), in which the value for the drug Gef and 17AAG were set to an initial state of 0 and 1 (reflecting either no treatment or standard treatment, respectively), and experimental nodes and mutations were adjusted (Table 3 and Box S2). All parameters for the proteins (‘nodes’) in the network without experimental or mutational regulation were set as an active node pulse (state = 0 and time = 0) that changes, depending on interconnectivity in the cell‐specific network.

#### 5.10.3 Software for visualization

To set up the silico topology we used the celldesigner software tool. For visualizing the network, we used cytoscape version 2.8.3 (Shannon *et al*., 2003). The cytoscape software is an open‐source platform for visualization and analysis of biological networks using several plug‐ins (Saito *et al*., 2012; Shannon *et al*., 2003). We analyzed the reconstructed cell line‐specific networks for functional modules (‘clusters’) using the Cytoscape plug‐in MCODE (Bader and Hogue, 2008). Potentially available drugs were selected using our previously developed DrumPID (Kunz *et al*., 2016).

The following methods were applied, as detailed below:

An *in silico* signaling network is invariably a simplified view of the biological complexity. We focus here on the major cascades relevant for the output. The bioinformatics analysis combined the cell culture array and western blot data with the systems biology network analysis approaches and information from databases. The robustness of the simplified networks of central tumor cascades in cell‐specific *in silico* networks was also verified by considering removing and adding protein nodes at the rim of the network. This did not affect signaling responses, whereas changing central hub protein nodes strongly affected the results. Cell compartmentalization (e.g., divergent cytosolic and mitochondrial processes), multiphosphorylation processes and complex formations were not included. This limits such approaches to semiquantitative descriptions of the sequential order, strength, and respective duration of events (Brietz *et al*., 2016; Göttlich *et al*., 2016). However, the simulations allow an *in silico* overview of the lung tumor topology and important drug responses, such as changes in individual cell line‐specific responses.

#### 5.10.4 Cell line‐specific network reconstruction

For establishing cell line‐specific signaling networks, we always used the same central cascades based on our previously published *in silico* topology (Göttlich *et al*., 2016; Stratmann *et al*., 2014) for HCC827 and A549 cell lines. In contrast, we extended the *in silico* network for the additionally introduced *KRAS*‐mutated H441 cell line.

#### 5.10.5 Simulation protocol

For dynamic simulation, we first looked at the effects of Gef and next 17AAG treatment in the tissue *in vitro* system, revealed by phospho‐RTK arrays and western blots. As the model is semiquantitative, weaker or stronger biological activation has to be taken into account with values between 0 (reflects no activation) and 1 (reflects full activation) to model key input. We fitted parameters to the results obtained from experiments (data‐driven modeling) and optimized the fit in iterative cycles of new simulations and new experiments. This included prestimulations according to mutations known from their pharmacological behavior. We hence simulated the proteins as network nodes with the parameters given in Table 3 and Box S2.

Additional information on the details of the bioinformatics analysis is given in the Doc. S1 and the Supporting information figures and boxes.

## Author contributions

CG performed all the experiments in the study, supported in some by SLN. MK performed all the bioinformatics work including simulations, network analysis, and large‐scale data analysis. In several places and in particular for the initial model set up, CZ worked on the bioinformatics analysis. GD and SLN supervised the experimental work. HW gave expert analysis and advice on all experimental work. HW, GD, and TD supervised CG, and TD and MK supervised CZ. TD and GD supervised the bioinformatics analysis, analyzed all data, and made suggestions for new experiments, simulations, data comparisons, and other analyses. TD and GD drafted the manuscript together, and all authors contributed to the iterations and agreed to the final version of the manuscript. TD and GD led and guided the study.

## Data availability

All data and simulation protocols for the study are made available with the publication (paper plus all Supporting information).

## Supporting information


**Fig. S1.** Signaling is unchanged in gefitinib responsive HCC827 cells in 2D and 3D.
**Fig. S2.** In silico model and simulation for the gefitinib treatment in A549 and H441.
**Fig. S3.** Signaling changes in 2D and 3D after treatment of different cell lines with the HSP90 inhibitor 17AAG.
**Fig. S4.** Biological network analyses on the KRAS‐mutated cell lines for 17AAG in the 3D system.
**Fig. S5.** Functional cluster analyses of the cell line‐specific networks.
**Fig. S6.** Cell line‐specific in silico simulations for gefitinib treatment in A549 and H441 according to data from the 2D system.
**Fig. S7.** In silico simulations for 17AAG treatment in A549 and H441 according to data from the 2D system.
**Box S1.** Ranking and comparison of all cell‐specific mutations for KRAS signature development and individual target predictions.
**Box S2.** Cell line‐specific differences modeled in 2D.

**Doc. S1.** Additional information for the bioinformatics analyses

**Table S1.** For the generation of networks we downloaded the HPRD which contains 9620 protein nodes and 39185 protein–protein interaction edges (release 9 from April 13, 2010).
**Table S2.** For the identification of a KRAS signature of potential markers we downloaded cell line‐specific mutations from the COSMIC database (A549: Sample Name: A549, Sample ID: 905949; H441: Sample Name: NCI‐H441, Sample ID: 908460).
**Table S3.** Mapping of the COSMIC mutations to the KRAS‐mutated network results in 18 H441‐ and 9 A549‐specific overlapping proteins (nodes).
