# Supplementary material for: A combined tissue‐engineered/in silico signature tool patient stratification in lung cancer
Source: Mol Oncol. 2018 Jun 22;12(8):1264–85. doi: 10.1002/1878-0261.12323 (PMC6068345; doi:10.1002/1878-0261.12323)
Supplement: Supplementary file 1 — Fig. S1. Signaling is unchanged in gefitinib responsive HCC827 cells in 2D and 3D. Fig. S2. In silico model and simulation for the gefitinib treatment in A549 and H441. Fig. S3. Signaling changes in 2D and 3D after treatment of different cell lines with the HSP90 inhibitor 17AAG. Fig. S4. Biological network analyses on the KRAS‐mutated cell lines for 17AAG in the 3D system. Fig. S5. Functional cluster analyses of the cell line‐specific networks. Fig. S6. Cell line‐specific in silico simulations for gefitinib treatment in A549 and H441 according to data from the 2D system. Fig. S7. In silico simulations for 17AAG treatment in A549 and H441 according to data from the 2D system. Box S1. Ranking and comparison of all cell‐specific mutations for KRAS signature development and individual target predictions. Box S2. Cell line‐specific differences modeled in 2D. [file MOL2-12-1264-s001.docx]

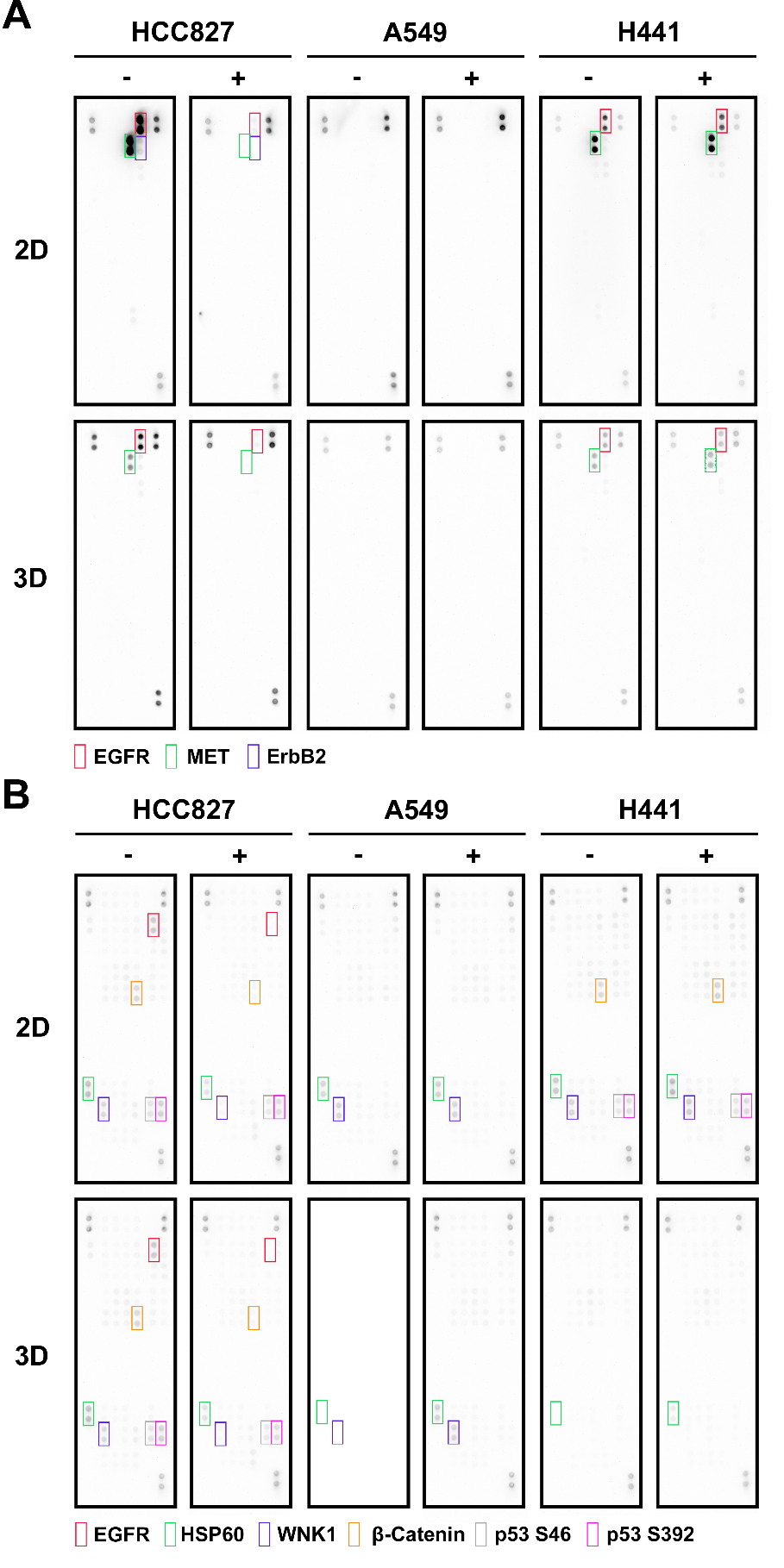


**Supplementary Figure S1: Signaling is unchanged in gefitinib responsive HCC827 cells in 2D and 3D.** (A) Phospho-RTK arrays of cell lysates of HCC827, A549 and H441 cultured in 2D and 3D, treated with 1 µM gefitinib for 72 h. The array contains 49 duplicated antibody spots for specific target receptor tyrosine kinases. Only the phosphorylated kinases were bound by the HRP-conjugated pan phospho-tyrosine antibody and visualized using chemiluminescence. (B) Phospho-Kinase arrays of cell lysates of HCC827, A549 and H441 cultured in 2D and 3D, treated with 1 µM gefitinib for 72 h. The array detects phosphorylation of 43 human kinases and total amount of two related proteins HSP60 and β-catenin.


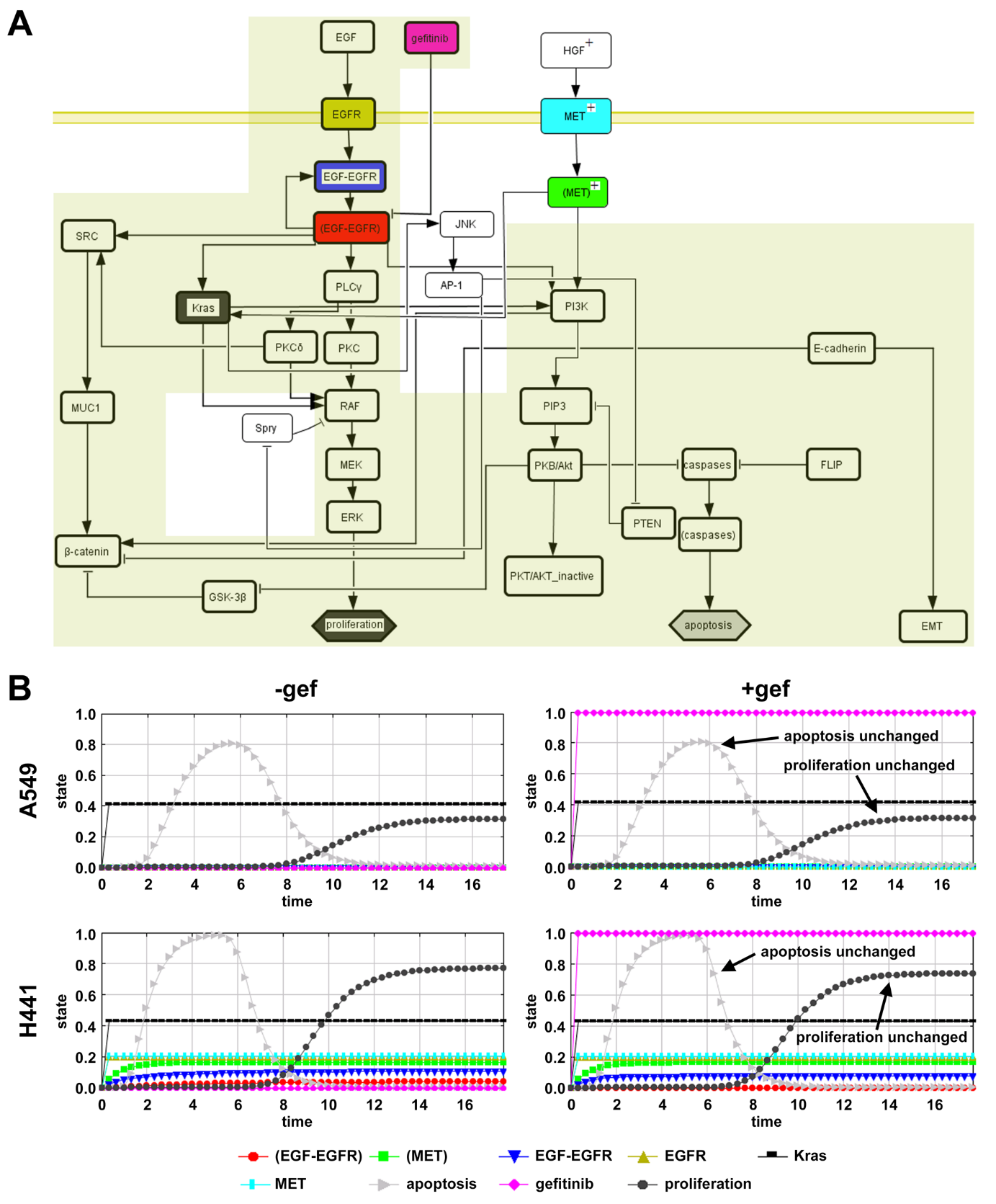


**Supplementary Figure S2: *In silico* model and simulation for the gefitinib treatment in A549 and H441.** (A) An *in silico* model of key pathways for the proliferative and apoptotic response upon gefitinib treatment was set up. Here a combined network for all cell lines is shown (Table 1 and 2 for regulated and included protein nodes). (B) Simulation of the gefitinib treatment (right, pink curve at stage 1) using the *in silico* model shows no differences in apoptosis (as grey curve) and proliferation (as black curve) compared to untreated cells (left, pink curve at level 0) for A549 (Top) and H441 (Bottom). For pre-stimulation see Table 3A.

**
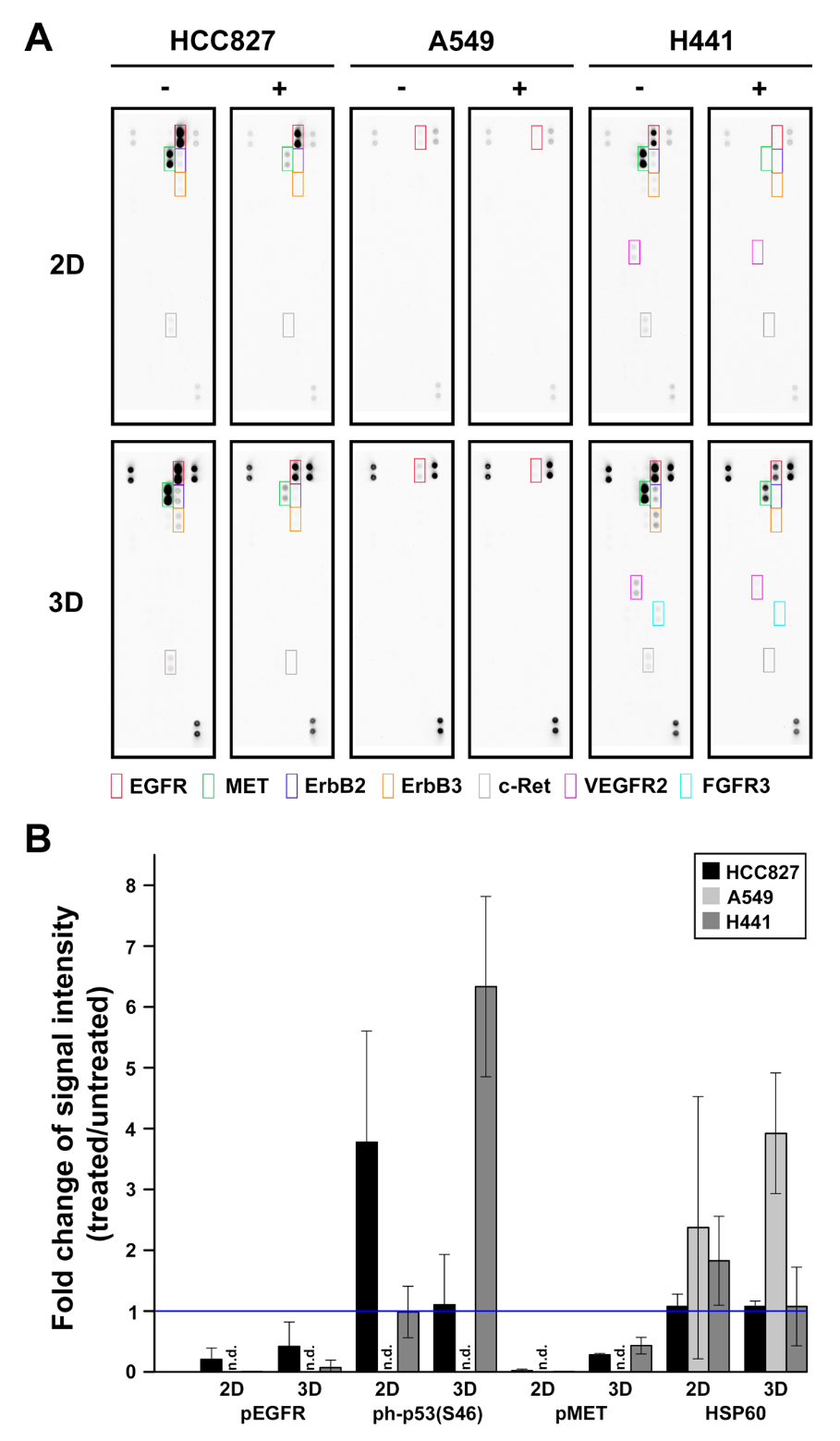
**

**Supplementary Figure S3: Signaling changes in 2D and 3D after treatment of different cell lines with the HSP90 inhibitor 17AAG.** (A) Phospho-RTK Arrays of cell lysates of HCC827, A549 and H441 cultured in 2D and 3D, treated with 0.25 µM 17AAG for 72 h. The array contains 49 duplicated antibody spots for specific target receptor tyrosine kinases. Only the phosphorylated kinases were bound by the HRP-conjugated pan phospho-tyrosine antibody and visualized using chemiluminescence. (B) Semi-quantitative Western blot analysis of cell lysates of HCC827, A549 and H441 cells treated with 17AAG for 72 h. Fold change of signal intensity of phosphorylated EGFR, p53 (S46), MET and HSP60 related to β-actin and control sample. n = 3, blue line labels the signal intensity of control samples that were set to 1.

**
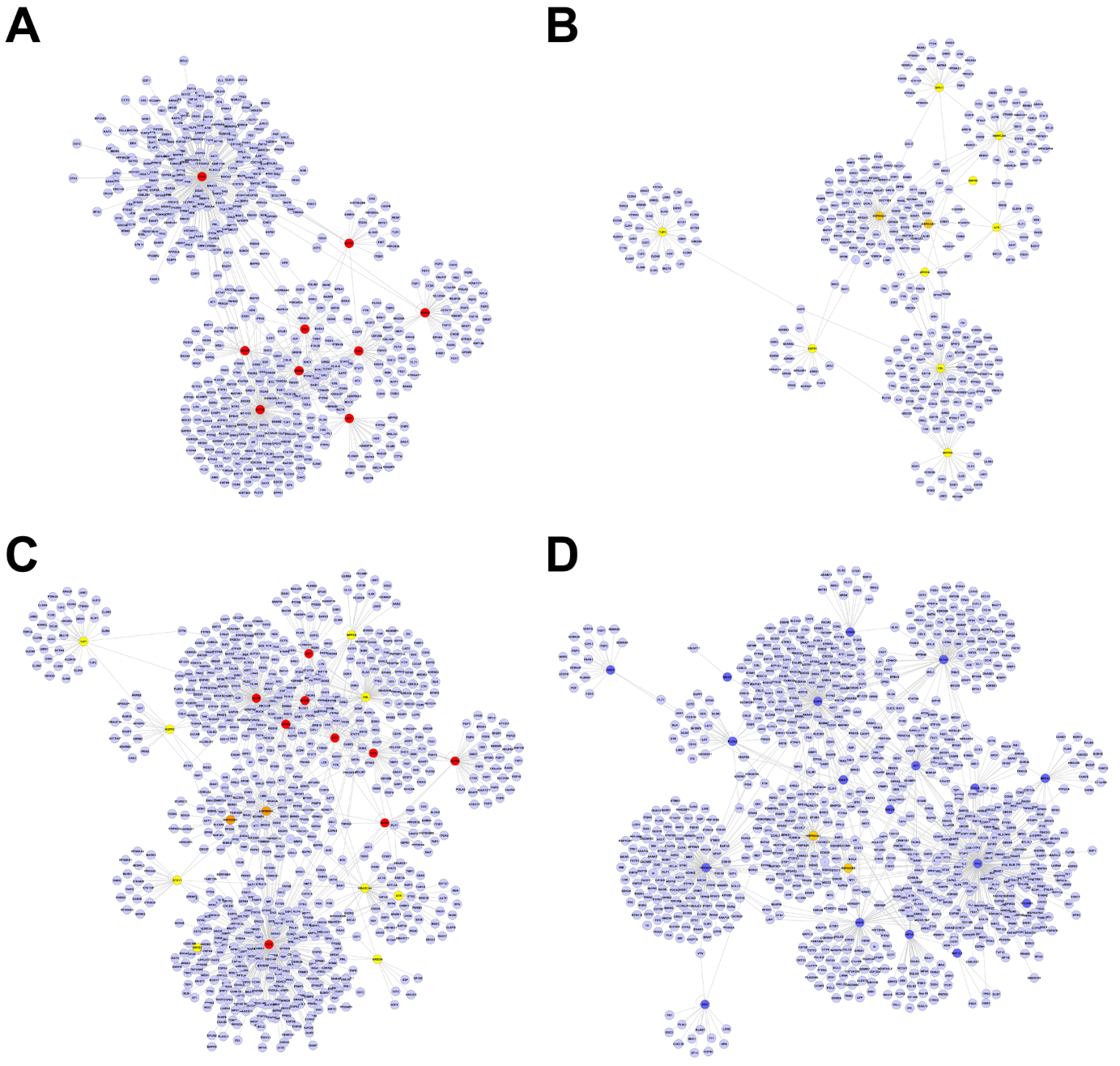
**

**Supplementary Figure S4: Biological network analyses on the *KRAS* mutated cell lines for 17AAG in the 3D system. (A)** Overview of the reconstructed KRAS interaction network. The reconstructed KRAS interaction network analyzed nine strongly deregulated proteins (DRP: EGFR, ErbB2, ErbB3, MET, FGFR3, c-Ret, VEGFR2, p53 and HSP60) upon 17AAG (Table 1) and their direct interaction partners from the HPRD database contains 556 protein nodes (DRPs in red, direct interaction partners in slate blue) and 680 interactions (grey lines). **(B) - (D)** We reconstructed cell line-specific networks based on the specific overlap between each cell and the network around KRAS (Venn diagram Figure 6a) and HSP90 as well as their direct interaction partners from the HPRD database. **(B)** The nine A549-specific mutations and HSP90 results in 322 protein nodes (nine mutations in yellow, HSP90AA1 and HSP90AB1 in orange, direct interaction partners in lavender) and 371 interactions (grey lines). **(C)** As the network size is small (e.g. compared to the H441 network from D), we combined the A549 network (C) with the KRAS interaction network (network A) which results in 795 nodes (DRPs in red, nine mutations in yellow, HSP90AA1 and HSP90AB1 in orange, direct interaction partners in lavender) and 1034 interactions (grey lines). **(D)** The 18 H441-specific proteins with mutations and HSP90 reconstitute a network of 903 protein nodes (18 mutations in blue, HSP90AA1 and HSP90AB1 in orange, direct interaction partners in lavender) and 1119 protein-protein interactions (grey lines).

**
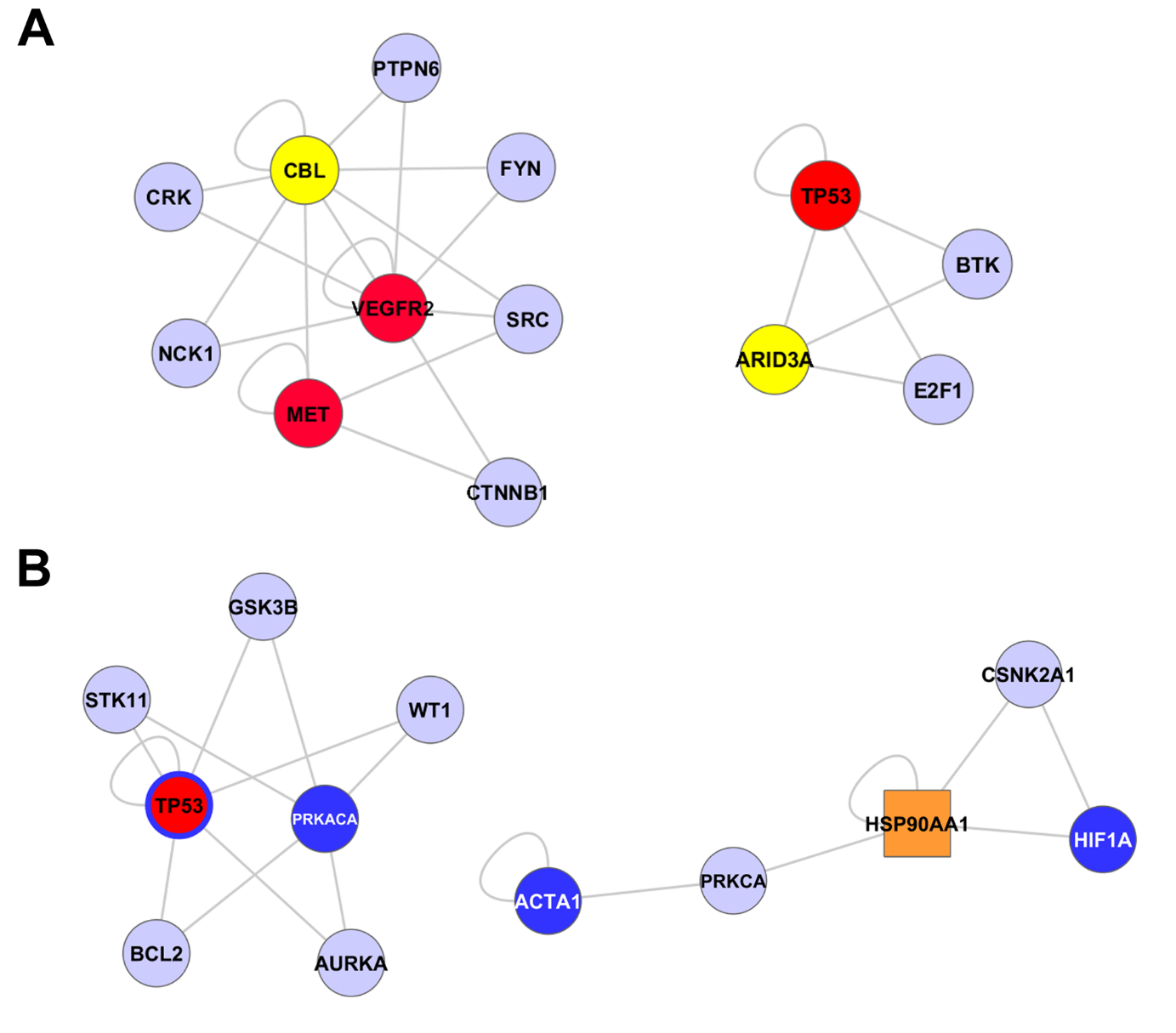
**

**Supplementary Figure S5: Functional cluster analyses of the cell line-specific networks.** Neighbor proteins are represented that we could target, if we consider the experimental data and directly interacting protein neighbors (from HPRD). **(A)** In the A549-specific network we found two functional clusters, one around VEGFR2, MET (both in red, experimental data) and CBL (ubiquitin ligase of tyrosine kinase receptors; cosmic data: A549 mutated, yellow) (Left), whereas the p53 signaling (red, experimental data) appears in one cluster with ARID3A (AT-Rich Interaction Domain 3A; cosmic data: A549 mutated, yellow) (Right). **(B)** In the H441-specific network the p53 signaling (red with blue circle, experimental data node and loss of function mutation according to COSMIC data) appears in one cluster with PRKACA (blue, Protein Kinase CAMP-Activated Catalytic Subunit Alpha; COSMIC data: H441 mutated), direct interactors shown in lavender (Left), whereas a second cluster lies around HSP90 (orange), HIF1A and ACTA (blue, H441 mutated according to COSMIC data), two direct interactors are shown in lavender (Right).

**
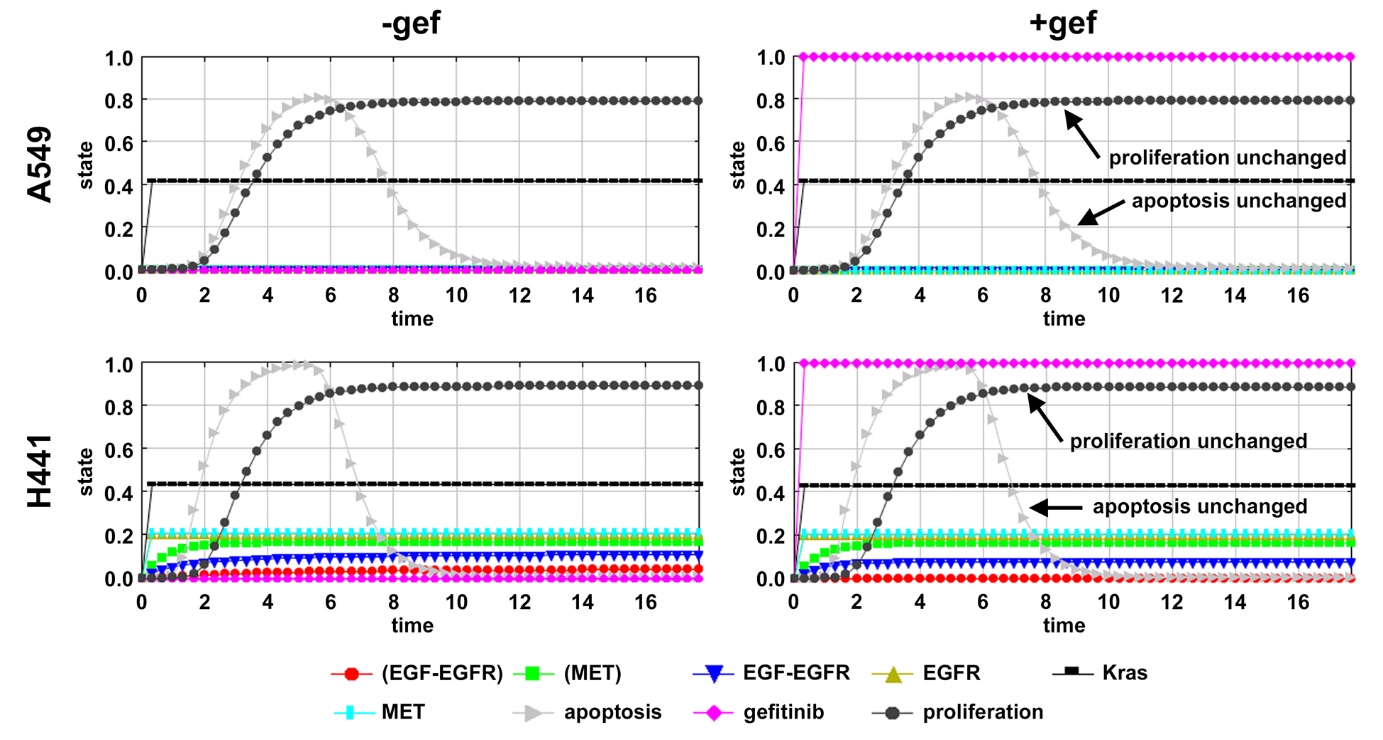
**

**Supplementary Figure S6: Cell line-specific *in silico* simulations for gefitinib treatment in A549 and H441 according to data from the 2D system.** Simulations of the gefitinib treatment reflect the *in vitro* data. Coloring of the curves is according to the network node colors shared for the cell lines shown in Supplementary Figure S2A. Cell line-specific pathway differences included are given in Table 1 and 2, pre-stimulation parameter in Supplementary box S2A. **(Top)** The *in silico* simulation of the gefitinib treatment in A549 cells (right, pink curve at full activation) shows no differences in apoptosis (grey curve) and proliferation (black curve) compared to untreated cells (left, pink curve at 0.0). However, A549 cells show a higher proliferation rate compared to the 3D model as it was observed *in vitro* (Supplementary Figure S2B). **(Bottom)** Simulation of the gefitinib treatment (right, pink curve full on at 1.0) for H441 shows no change in apoptosis (grey curve) and proliferation (black curve) compared to untreated cells (left, pink curve at 0.0). The proliferation rate correlates with the *in vitro* data.

**
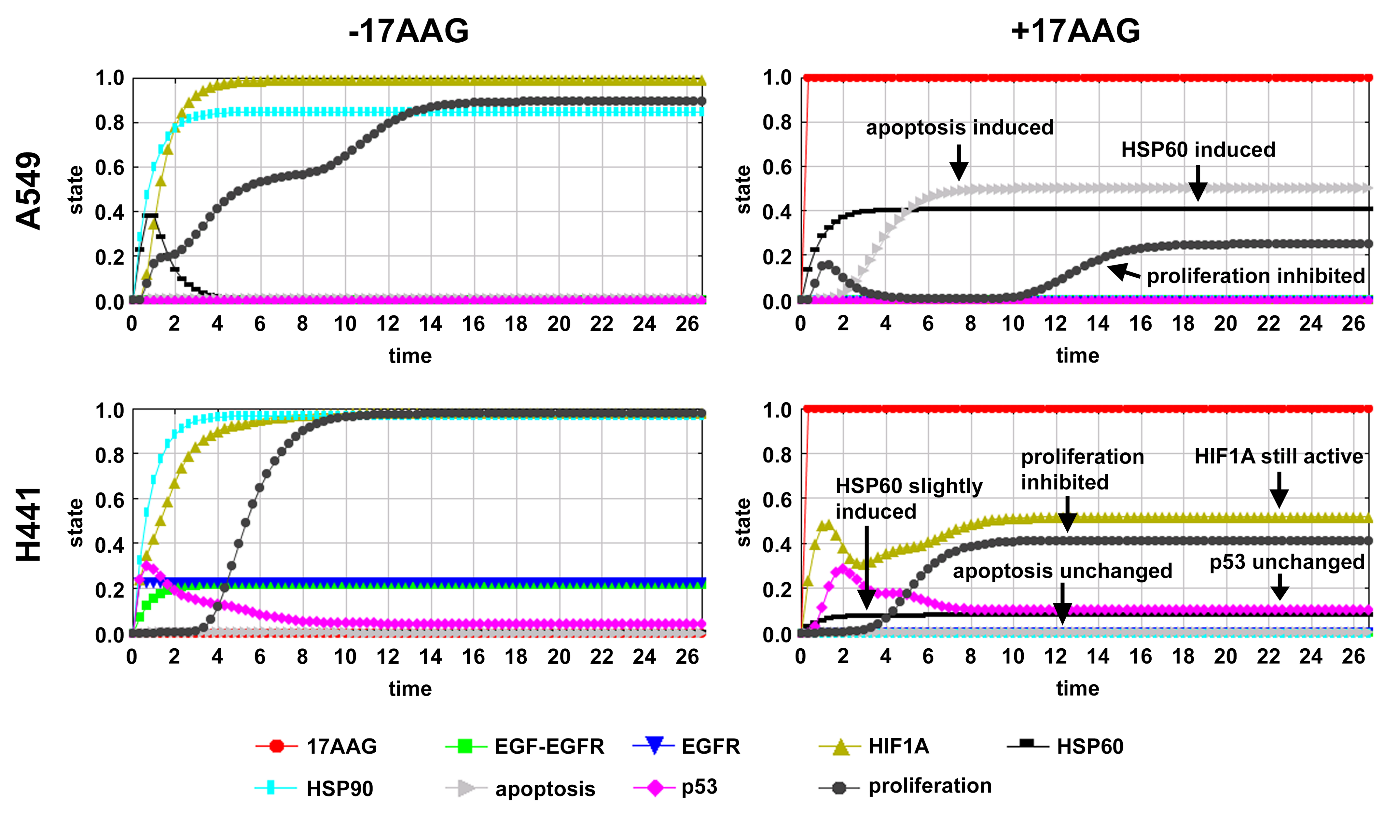
**

**Supplementary Figure S7: *In silico* simulations for 17AAG treatment in A549 and H441 according to data from the 2D system.** Simulations of the 17AAG treatment reflect the *in vitro* data. Coloring of the curves is according to the network node colors shared for the cell lines shown in Figure 4B. Cell line-specific pathway differences included are given in Table 1 and 2, pre-stimulation parameter in Supplementary box S2B. **(Top)** Simulation of the 17AAG treatment in A549 cells (right, red curve at 1) results in strong apoptosis induction (grey curve) and reduced proliferation (black curve) as compared to untreated cells (left, red curve at 0.0). The simulations shows a weaker induction of HSP60 (black curve) in 2D compared to 3D. **(Bottom)** In H441 cells, apoptosis is unchanged (grey curve), whereas the proliferation (black curve, dots) is inhibited. However, compared to 3D, p53 (pink curve) is unchanged, and HSP60 (black curve, squares) is slightly induced after 17AAG treatment and correlates with the *in vitro* data.

**Supplementary box S1: Ranking and comparison of all cell-specific mutations for KRAS signature development and individual target predictions.** Based on our integrated systems biology analysis we performed an *in silico* screen and ranked targets related to HSP90AA1 and HSP90AB1, and the DRPs from the array using our DrumPID database (Kunz, Liang et al. 2016). Regarding ranking of drug targets for potential clinical application, we considered proteins and connections and assigned the priority (i) to direct neighbors of the central cascade, (ii) if they are mutated (driver mutations) and (iii) could be targeted easily by existing medical drugs, e.g., AMPK for A549 and HIF1A for H441. The top criterion dominates the second and this the third criterion.

**A549**

Top Rank:

- LKB1 (mutated), AMPK (interactor of p53 (array, cluster node; HSP90AA1/HSP90AB1))

Rank 2:

- SMARCA4 (mutated) + interactor of LKB1 (mutated; top rank) + p53 (array, cluster node; interacts with HSP90AA1/HSP90AB1)

- ARID3A (mutated, cluster node) + interactor of p53 (array, cluster node; interacts with HSP90AA1/HSP90AB1)

- CBL (mutated, cluster node) + interactor of VEGFR2 (array, cluster node; interacts with HSP90AA1) + MET (array, cluster node) + EGFR (array) + RET (array)

- CSNK2A1 (interactor showing no mutation) + interactor of p53 (array, cluster node; interacts with HSP90AA1/HSP90AB1) + HSP90AA1

- NR3C1 (interactor showing no mutation) + interactor of p53 (array, cluster node; interacts with HSP90AA1/HSP90AB1) + HSP90AA1

Rank 3:

- SMYD2 (mutated) + interactor of p53 (array, cluster node; interacts with HSP90AA1/HSP90AB1)

- ATR (mutated) + interactor of p53 (array, cluster node; interacts with HSP90AA1/HSP90AB1) + interactor of VEGFR2 (array, cluster node; interacts with HSP90AA1)

- INPP5D (mutated) + interactor of MET (array, cluster node)

- AGTR1 (mutated) + interactor of EGFR (array)

- TJP1 (mutated) + interactor of EGFR (array)

Rank 4:

- PPP5C (mutated and interactor of one mutated gene)

- APOB (mutated and interactor of one mutated gene)

- CD2AP (mutated and interactor of one mutated gene)

- CTNNA1 (mutated and interactor of one mutated gene)

- THOP1 (mutated and interactor of one mutated gene)

- FLT3 (mutated and interactor of one mutated gene)

Rank 5:

- BTK (interactor showing no mutation, cluster node) + interactor of p53 (array, cluster node; interacts with HSP90AA1/HSP90AB1) + CBL (mutated, cluster node) + ARID3A (mutated, cluster node)

- FYN (interactor showing no mutation, cluster node) + interactor of VEGFR2 (array, cluster node; interacts with HSP90AA1) + CBL (mutated, cluster node) and HSP90AA1

- E2F1 (interactor showing no mutation, cluster node) + interactor of p53 (array, cluster node; interacts with HSP90AA1/HSP90AB1) + ARID3A (mutated, cluster node) + ATR (mutated)

- PTPN6 (interactor showing no mutation, cluster node) + interactor of VEGFR2 (array, cluster node; interacts with HSP90AA1) + CBL (mutated, cluster node) + interactor of EGFR (array)

- SRC (interactor showing no mutation, cluster node) + interactor of CBL (mutated, cluster node) + interactor of VEGFR2 (array, cluster node; interacts with HSP90AA1) + MET (array, cluster node) + EGFR (array) + ERBB2 (array) + RET (array) + HSP90AA1

- CRK (interactor showing no mutation, cluster node) + CBL (mutated, cluster node) + interactor of VEGFR2 (array, cluster node; interacts with HSP90AA1) + EGFR (array)

- CTNNB1 (interactor showing no mutation, cluster node) + interactor of VEGFR2 (array, cluster node; interacts with HSP90AA1) + MET (array, cluster node) + ERBB2 (array) + SMARCA4 (mutated)

- NCK1 (interactor showing no mutation, cluster node) + CBL (mutated, cluster node) + interactor of VEGFR2 (array, cluster node; interacts with HSP90AA1) + EGFR (array)

- RASA1 (interactor showing no mutation) + interactor of HSP60 (array; interacts with HSP90AA1/HSP90AB1, p53) + VEGFR2 (array, cluster node; interacts with HSP90AA1) + EGFR (array)

- ATF3 (interactor showing no mutation) + interactor of p53 (array, cluster node; interacts with HSP90AA1/HSP90AB1) + FGFR3 (array)

- PTK2B (interactor showing no mutation) + interactor of FGFR3 (array) + ERBB2 (array) + ERBB3 (array) + EGFR (array)

**H441**

Top Rank:

- HIF1A (mutated, cluster node) + interacts with p53 (array, mutated, cluster node, interacts with HSP90AA1/HSP90AB1) + HSP90AA1 (cluster node) + CSNK2A1 (inter, cluster node)

Rank 2:

- PRKCA (interactor showing no mutation, cluster node) + interacts with p53 (array, mutated, cluster node, interacts with HSP90AA1/HSP90AB1) + interacts with EGFR (array, mutated) + HSP90AA1 (cluster node)

- CSNK2A1 (interactor showing no mutation, cluster node) + HIF1A (mutated, cluster node) + interacts with p53 (array, mutated, cluster node, interacts with HSP90AA1/HSP90AB1) + HSP90AA1 (cluster node) + TOP2A (mutated)

- NR3C1 (interactor showing no mutation) + interacts with p53 (array, mutated, cluster node, interacts with HSP90AA1/HSP90AB1) + HSP90AA1 (cluster node) + PRKACA (mutated, cluster node)

- ACTA1 (mutated, cluster node) + interacts with p53 (array, mutated, cluster node, interacts with HSP90AA1/HSP90AB1)

- PRKACA (mutated, cluster node) + interacts with EGFR (array, mutated) + NOS1 (interacts with HSP90AA1) + NR3C1 (interacts with HSP90AA1 (cluster node), HSP60 (array; interacts with HSP90AA1/HSP90AB1, p53) and p53 (array, mutated, cluster, interacts with HSP90AA1/HSP90AB1))

- NOS1 (mutated and interactor of one mutated gene) + interacts with HSP90AA1 (cluster node) + PRKACA (mutated, cluster node; interacts with EGFR (array, mutated)

- IKBKE (mutated and interactor of one mutated gene) + interacts with HSP90AA1 (cluster node) + HSP90AB1

Rank 3:

- KAT5 (mutated) + interacts with p53 (array, mutated, cluster node, interacts with HSP90AA1/HSP90AB1)

- TP53INP1 (mutated) + interacts with p53 (array, mutated, cluster node, interacts with HSP90AA1/HSP90AB1)

- NAP1L1 (mutated) + interacts with p53 (array, mutated, cluster node, interacts with HSP90AA1/HSP90AB1)

- HTT (mutated) + interacts with p53 (array, mutated, cluster node, interacts with HSP90AA1/HSP90AB1) + EGFR (array, mutated)

- TOP2A (mutated) + interacts with p53 (array, mutated, cluster node, interacts with HSP90AA1/HSP90AB1) + HSP90AA1 (cluster node)

- WWOX (mutated) + interacts with p53 (array, mutated, cluster node, interacts with HSP90AA1/HSP90AB1)

- BRCA2 (mutated) + interacts with p53 (array, mutated, cluster node, interacts with HSP90AA1/HSP90AB1)

- ERBB4 (mutated) + interacts with ERBB3 (array) + ERBB2 (array) + EGFR (array, mutated)

Rank 4:

- IQGAP1 (mutated) + interacts with VEGFR2 (array)

- MUC4 (mutated) + interacts with ERBB2 (array)

- PLCG2 (mutated) + interacts with VEGFR2 (array)

- NRP1 (mutated) + interacts with VEGFR2 (array)

- HGF (mutated) + interacts with MET (array)

Rank 5:

KLKB1 (mutated and interactor of one mutated gene)

TNC (mutated and interactor of one mutated gene)

DAPP1 (mutated and interactor of one mutated gene)

IK (mutated and interactor of one mutated gene)

TTN (mutated and interactor of one mutated gene)

SI (mutated and interactor of one mutated gene)

TNNI3K (mutated and interactor of one mutated gene)

SLC2A2 (mutated and interactor of one mutated gene)

SYMPK (mutated and interactor of one mutated gene)

MYO7A (mutated and interactor of one mutated gene)

RYR2 (mutated and interactor of one mutated gene)

APOB (mutated and interactor of one mutated gene)

GRIA1 (mutated and interactor of one mutated gene)

FAM135B (mutated and interactor of one mutated gene)

PALB2 (mutated and interactor of one mutated gene)

Rank 6:

- LKB1 (interactor showing no mutation, cluster node) + interacts with p53 (array, mutated, cluster node, interacts with HSP90AA1/HSP90AB1) + PRKACA (mutated, cluster node, interacts with EGFR (array, mutated))

- BCL2 (interactor showing no mutation, cluster node) + interacts with p53 (array, mutated, cluster node, interacts with HSP90AA1/HSP90AB1) + PRKACA (mutated, cluster node, interacts with EGFR (array, mutated))

- WT1 (interactor showing no mutation, cluster node) + interacts with p53 (array, mutated, cluster node, interacts with HSP90AA1/HSP90AB1) + PRKACA (mutated, cluster node, interacts with EGFR (array, mutated))

- GSK3B (interactor showing no mutation, cluster node) + interacts with p53 (array, mutated, cluster node, interacts with HSP90AA1/HSP90AB1) + PRKACA (mutated, cluster node, interacts with EGFR (array, mutated))

- AURKA (interactor showing no mutation, cluster node) + interacts with p53 (array, mutated, cluster node, interacts with HSP90AA1/HSP90AB1) + PRKACA (mutated, cluster node, interacts with EGFR (array, mutated))

- CBL (interactor showing no mutation) + interactor of RET (array) + VEGFR2 (array)

- ATF3 (interactor showing no mutation) + interactor of p53 (array, mutated, cluster node, interacts with HSP90AA1/HSP90AB1) + FGFR3 (array)

- PTK2B (interactor showing no mutation) + interactor of FGFR3 (array) + ERBB2 (array) + ERBB3 (array) + EGFR (array, mutated)

**- Supplementary box S2:** Cell line-specific differences modeled in 2D

**- Supplementary box S2:** Cell line-specific differences modeled in 2D

**Supplementary box S2: Cell line-specific differences modeled in 2D**

**A) Cell line-specific differences in pathway activities on gefitinib^1^**

| cell-line | Parameter | (-) gef | (+) gef |
| --- | --- | --- | --- |
| A549 (2D) | KRAS^#^ | 0.413 | 0.413 |
|  | FLIP | 0.6 | 0.6 |
|  | RAF | 0.27 | 0.27 |
| H441 (2D) | KRAS^#^ | 0.43 | 0.43 |
|  | EGFR | 0.205 | 0.205 |
|  | MET | 0.205 | 0.205 |
|  | FLIP | 0.4 | 0.4 |
|  | RAF | 0.28 | 0.28 |

**B) Cell line-specific differences in pathway activities on 17AAG^1^**

| cell-line | parameter | (-) 17AAG | (+) 17AAG |
| --- | --- | --- | --- |
| A549 (2D) | KRAS^#^ | 0.352 | 0.352 |
|  | Stress | 0.5 | 0.5 |
|  | FLIP | 0.7 | 0.0 |
|  | p53 | 0.0 | 0.0 |
|  | HSP60-act | 0.4 | 0.3 |
|  | RAF | 0.28 | 0.08 |
| H441 (2D) | KRAS^#^ | 0.43 | 0.43 |
|  | EGFR | 0.23 | 0.01 |
|  | Erb2/Erb3/c-RET/FGFR3 | 0.04 | 0.03 |
|  | MET | 0.23 | 0.01 |
|  | Stress | 0.7 | 0.7 |
|  | p53-act | 0.65 | 0.01 |
|  | HIF1-act | 0.65 | 0.65 |
|  | HSP60-act | 0.05 | 0.15 |
|  | VEGFR2 | 0.35 | 0.33 |
|  | FLIP | 0.75 | 0.75 |
|  | PTEN | 0.41 | 0.41 |
|  | RAF | - | 0.22 |

**^1^** Cell line-specific receptor or pathway activity of proteins according to the experimentally determined differences in response behavior (apoptosis, proliferation, RTK and Western blot data); all other nodes were modeled with no specific activation. (-) treatment activation at stage 0, (+) treatment activation at stage 0. The hash (#) indicates constant activation, as there is a *KRAS* mutation in these cell lines. Differences to the 3D simulation activities (Table 3A,B) are labeled in red.
