# Supplementary material for: A combined tissue‐engineered/in silico signature tool patient stratification in lung cancer
Source: Mol Oncol. 2018 Jun 22;12(8):1264–85. doi: 10.1002/1878-0261.12323 (PMC6068345; doi:10.1002/1878-0261.12323)
Supplement: Supplementary file 2 — Doc. S1. Additional information for the bioinformatics analyses [file MOL2-12-1264-s002.docx]

**Additional information for the bioinformatics analyses**

Our approach is generic and can be systematically applied to examine cell line-specific responses in cancer and suggesting tailored therapies for precision medicine, in practice by mapping the patient-specific mutations after sequencing on a cell line-specific *in silico* model. To show how this is done in practice we give here more information on the specific bioinformatics steps involved:

(i) details on the network reconstruction for *KRAS* mutated cell lines in the 3D system and identification of new biomarker signatures and drug targets.

(ii) Furthermore, we explain details on the calculated cell-specific signatures given in Table 3 in the results.

(iii) There is also more information on our tool regarding therapeutic, cell-specific targeting.

**(i) Network reconstruction for *KRAS* mutated cell lines in the 3D system and identification of new biomarker signatures and drug targets**

To identify a signature of potential biomarkers and drug targets, we combined quantitative kinase and protein expression changes with high confidence human interactome data from HPRD and genomic mutational data from COSMIC for systems biology analysis. The overall analysis approach follows our developed systems biology network method (Naseem, Kunz et al. 2014) (Kunz, Dandekar et al. 2017). As a first step, we selected Differentially Regulated Proteins (DRPs) between both cell lines in the 3D *in vitro* system (Table 1). The resulting nine DRPs were used to reconstruct a KRAS network: we downloaded for this all direct binary human protein-protein interactions (PPIs) from the HPRD (9620 proteins (“nodes”) and 39185 protein-protein interactions (“edges”), release 9 from April 13, 2010; Table S1 in Supplementary excel file) and warehoused them in a MySQL database. As a next step, we mapped the selected DRPs against all interactions in our database to reconstruct the KRAS sub-network considering only direct neighbors, whereby we see the interplay between all proteins. This network contains 556 proteins (“nodes”) and 680 protein-protein interactions (“edges”; see Supplementary Figure S4A). As shown in the figure, neighbor proteins interact with the central cascades and have 1^st^ and 2^nd^ degree neighbors. We next looked for directly interacting human proteins and revealed by this additional drug targets to counter the KRAS resistance mimicked here by specific mutations in the two cell lines A549 and H441. To achieve this, we used cell line-specific mutations from the COSMIC database (A549: Sample Name: A549, Sample ID: 905949, 361 mutations; H441: Sample Name: NCI-H441, Sample ID: 908460, 573 mutations; Supplementary Table S2 in Supplementary excel file) and mapped them to the 556 proteins (nodes) from the KRAS network. The Venn diagram represents specific overlaps between H441 (blue), A549 (yellow) and the KRAS proteins (green), indicating 18 H441-specific mutations and nine A549-specific mutations (see Figure 6A in the paper; Supplementary Table S3 in Supplementary excel file for the overlapping mutations). We next reconstructed with this individual overlapping protein nodes and HSP90 and their directly interacting partners from HPRD for each cell line, A549 and H441, a cell line-specific network and performed functional cluster analysis. The A549-specific network contains 322 protein nodes and 371 protein-protein interaction edges (Supplementary Figure S4B). As the network size is small (also compared to the H441 network), we decided to combine it with the KRAS network from Supplementary Figure S4A. This combined network contains 795 protein nodes and 1034 protein-protein interaction edges (Supplementary Figure S4C), in which we found two functional clusters around VEGFR2, MET and CBL (score=1.667; in yellow) as well as p53 and ARID3A (Score=1.25; in yellow; Supplementary Figure S5A). Similarly, using the 18 overlapping nodes for H441 and HSP90 and their directly interacting partners from HPRD we reconstructed a H441-specific network (903 nodes and 1119 edges; Supplementary Figure S4D). Here we found two clusters around PRKACA and p53 (score=1.429; in blue) as well as HSP90AA1, ACTA and HIF1A (Score=1; found in orange in Figure S5B). Finally, integrating the A549-specific mutations (in yellow), HSP90AA1 and HSP90AB1 (in orange), the cluster protein nodes (non A549-mutated/non DRPs in lavender) and DRPs from the array (in red) results in a highly connected signature sub-network of 40 protein nodes and 89 shared protein-protein interaction edges (Figure 6B in the paper). Notably, additional six 2^nd^ interacting A549 mutations (in cyan) and connections between interesting drivers in tumor progression which are linked directly or through one step (by-standers) to the nodes become much clearer.

Integration of all information for H441 (18 H441-specific mutations (in blue), HSP90AA1 and HSP90AB1 (in orange), the cluster protein nodes (non H441-mutated/non DRPs in lavender) and DRPs from array (in red; p53 and EGFR in red and blue as both are array nodes and mutated) shows a highly connected sub-network of 55 protein nodes and 92 protein-protein interaction edges, whereby 17 additional 2^nd^ interacting proteins in H441 cells with mutations (in cyan) become apparent (Figure 6C in the paper).

This allows now to screen *in silico* for promising protein targets among these protein neighbors using our DrumPID database (Kunz, Liang et al. 2016), as targeting these proteins should influence cancer proliferation and apoptosis (Supplementary box S1). Finally, the potential therapeutic effect on apoptosis and proliferation for the two identified targets AMPK (A549) and HIF1A (H441) as top ranked candidates from the drug screening were dynamically simulated using the same steps as described above.

**(ii) Details on the calculated cell-specific signatures given in Table 4 in results:**

Applying integrated systems biology analysis by considering the experimental data and the mutational background, we identified several cell line-specific predictive markers and new therapeutic targets for the A549 and H441 cell lines. The table represents a signature of important markers and drug targets, which lie in functional protein clusters and/or connected to HSP90. These protein nodes might represent promising predictive marker for a 17AAG treatment and alternative therapeutic drug targets (in blue reflects the mutational status in each cell line, whereas orange indicates unknown mutational status in both cell lines; wt=wildtype). The Serine-Threonin-Kinase (STK) Inhibitor Of Kappa Light Polypeptide Gene Enhancer In B-Cells, Kinase Epsilon (IKBKE) is a newly identified oncogene which is activated by STAT3 and smoke, thus might function as potential marker and target for smoking-associated NSCLC (Guo, Kim et al. 2013). The Nitric Oxide Synthase 1 (NOS1) is linked to HSP90. Nitric synthases are known to be associated with tumor pathogenesis and show deregulation in NSCLC. Studies reported a higher NOS1 level as a good prognostic marker, whereas NOS2 favours KRAS induced lung tumors (Puhakka, Kinnula et al. 2003) (Puhakka, Harju et al. 2006) (Okayama, Saito et al. 2013). For A549 we identified the tumor suppressor BRG1 (SWI/SNF Related, Matrix Associated, Actin Dependent Regulator Of Chromatin, Subfamily A, Member 4; synonym: SMARCA4) as potential marker and target. It functions as important factor in tumorigenesis and is associated with bad prognosis in NSCLC, in which a co-mutation e.g. of *KRAS* and *LKB1* is observed (Medina, Romero et al. 2008) (Reisman, Sciarrotta et al. 2003). Moreover, SMARCA is a novel prognostic marker with higher sensitivity against platin-based chemotherapies in NSCLC (Bell, Chakraborty et al. 2016). Casitas B-lineage lymphoma (Cbl) is altered in lung cancer and functions as new therapeutic target in lung cancer (Tan, Krishnaswamy et al. 2010), in which studies shown for A549 that ectopic c-Cbl expression reduce tumor growth *in vitro* and tumor metastasis in xenograft models (Lo, Tan et al. 2011) (Wei, Lin et al. 2015). For CSNK2A1 and NR3C1 there are no information in COSMIC available about their mutational status in A549 and H441, thus they can function as marker and target for both *KRAS* mutated NSCLC cell lines. The Casein Kinase 2, Alpha 1 Polypeptide (CSNK2A1) is overexpressed in various tumors and is associated with apoptosis, survival and the PI3K/AKT-signaling pathway. The identified chromatin remodeling Nuclear Receptor Subfamily 3, Group C, Member 1 (NR3C1) is encoding the glucocorticoid receptor and linked e.g. to p53 and HSP90, however knowledge in lung cancer is limited.

**(iii) Therapeutic, cell-specific targeting and *in silico* modeling**

**Exemplified target and drug candidate identification for A549 cells:** we selected LKB1 as relevant target candidate for A549 as it is mutated and an interactor of p53 (array/Western blot node) which is in a functional cluster and interacting with HSP90AA1 and HSP90AB1 (Figure 6B in the paper and S5A in the supplement). AMPK becomes phosphorylated and activated by LKB1 during energy depletion and regulates cellular processes such as glucose transport and cell cycle arrest. Inactivation of AMPK favors cell proliferation and growth through the mTOR signaling pathway. As neither an FDA-approved nor a non-FDA-approved drug is available for LKB1 we decided to select its downstream gene AMPK, which is also a better strategy as this gene is only influenced but not directly mutated. For AMPK we found the activators AICAR (5-Aminoimidazole-4-carboxamide ribonucleotide) and Phenformin (search AMPK in DrumPID database, pathway ko04152 shows AMPK interactome in detail). However, we selected AICAR as first best selective AMPK activator, which is also under clinical investigation for cancer. However, other activators are not under clinical trial, or they are for different interventions, e.g. Phenformin is an anti-diabetic drug, and at present no data are available on its anti-cancer efficiency as a consortium just started recruiting patients for BRAF-mutated melanoma in Jan 2017 (for more information see Selleckchem link in DrumPID). In contrast, therapeutic activation of AMPK is currently investigated for various cancers. AICAR inhibits *in vitro* and *in vivo* tumor growth as was also reported for A549 cell lines (Rattan, Giri et al. 2005) (Fay, Steele et al. 2009) (Tang, Williams et al. 2011).

**Exemplified target and drug candidate identification for H441 cells:** for H441, HIF1A is our top candidate as it is mutated and forms a functional cluster with HSP90AA1, but also interacts with p53 (array/Western blot node and mutated). P53 is also part of a functional cluster (Figure 6C in the paper and Figure S5B in the supplement). HIF1A regulates angiogenesis and proliferation (Ravi, Mookerjee et al. 2000) (Greijer and van der Wall 2004). It leads to higher mortality in various cancers including NSCLC (Semenza 2003). For HIF1A we found as drug candidates e.g. BAY 87-2243, PX-478, 2-Methoxyestradiol (2-MeOE2) and Carvedilol (see HIF1A in DrumPID, HIF1A network for detail). We selected PX-478 as first best selective HIF1A inhibitor, which is also under clinical trial for cancer. The other activators are either not selective or not under clinical trial for cancer, e.g. BAY 87-2243 is used in a phase 1 trial for Neoplasms, 2-Methoxyestradiol (2-MeOE2) is investigated in a clinical trial for cancer but no selective HIF1A inhibitor and Carvedilol is not a selective HIF1A inhibitor and investigated as drug for heart diseases (see also Selleckchem link in DrumPID). Interestingly, HIF1A shows first promising therapeutic effects in human xenograft models (Welsh, Williams et al. 2004). PX-478 is also under clinical investigation (phase 1) in prostate cancer (Palayoor, Mitchell et al. 2008).

Subsequently, such targets and drugs can be integrated in the *in silico* network by considering its specific connectivity to the central cascades. Further *in silico* simulations with SQUAD investigate their potential therapeutic effect on apoptosis and proliferation. Here, we focus on AICAR and PX-478 as top candidates, but our tool also allows integrating other drug target candidates by considering individual targets and side-targets. However, an iterative cycle of *in silico* simulation and further experimental testing of predictions helps in finding the best targets with clinical relevance.

**Shared drug target candidates from our *KRAS* signature**

In addition, we identified some promising drug target structures occurring in both cell lines (Supplementary box S1). Such a promising target is CSNK2A1. Experimental studies of Casein-Kinase-2 inhibitors demonstrated first therapeutic success in NSCLC (Zhang, Long et al. 2013) (So, Rho et al. 2015). For NR3C1 first studies reported a correlation with lung cancer progression (Zhao, Liu et al. 2015) (Geng, Zhu et al. 2016). Cortisone, dexamethasone and prednisone are potential NR3C1 targeting drugs (identified from our DrumPID database) clinically tested for cancer (e.g. leukemia and prostate cancer).

**References**

Bell, E. H., A. R. Chakraborty, X. Mo, Z. Liu, K. Shilo, S. Kirste, P. Stegmaier, M. McNulty, N. Karachaliou, R. Rosell, G. Bepler, D. P. Carbone and A. Chakravarti (2016). "SMARCA4/BRG1 Is a Novel Prognostic Biomarker Predictive of Cisplatin-Based Chemotherapy Outcomes in Resected Non-Small Cell Lung Cancer." Clin Cancer Res **22**(10): 2396-2404.

Fay, J. R., V. Steele and J. A. Crowell (2009). "Energy homeostasis and cancer prevention: the AMP-activated protein kinase." Cancer Prev Res (Phila) **2**(4): 301-309.

Geng, L., M. Zhu, Y. Wang, Y. Cheng, J. Liu, W. Shen, Z. Li, J. Zhang, C. Wang, G. Jin, H. Ma, H. Shen, Z. Hu and J. Dai (2016). "Genetic variants in chromatin-remodeling pathway associated with lung cancer risk in a Chinese population." Gene **587**(2): 178-182.

Greijer, A. E. and E. van der Wall (2004). "The role of hypoxia inducible factor 1 (HIF-1) in hypoxia induced apoptosis." J Clin Pathol **57**(10): 1009-1014.

Guo, J., D. Kim, J. Gao, C. Kurtyka, H. Chen, C. Yu, D. Wu, A. Mittal, A. A. Beg, S. P. Chellappan, E. B. Haura and J. Q. Cheng (2013). "IKBKE is induced by STAT3 and tobacco carcinogen and determines chemosensitivity in non-small cell lung cancer." Oncogene **32**(2): 151-159.

Kunz, M., T. Dandekar and M. Naseem (2017). "A Systems Biology Methodology Combining Transcriptome and Interactome Datasets to Assess the Implications of Cytokinin Signaling for Plant Immune Networks." Methods Mol Biol **1569**: 165-173.

Kunz, M., C. Liang, S. Nilla, A. Cecil and T. Dandekar (2016). "The drug-minded protein interaction database (DrumPID) for efficient target analysis and drug development." Database (Oxford) **2016**.

Lo, F. Y., Y. H. Tan, H. C. Cheng, R. Salgia and Y. C. Wang (2011). "An E3 ubiquitin ligase: c-Cbl: a new therapeutic target of lung cancer." Cancer **117**(23): 5344-5350.

Medina, P. P., O. A. Romero, T. Kohno, L. M. Montuenga, R. Pio, J. Yokota and M. Sanchez-Cespedes (2008). "Frequent BRG1/SMARCA4-inactivating mutations in human lung cancer cell lines." Hum Mutat **29**(5): 617-622.

Naseem, M., M. Kunz and T. Dandekar (2014). "Probing the unknowns in cytokinin-mediated immune defense in Arabidopsis with systems biology approaches." Bioinform Biol Insights **8**: 35-44.

Okayama, H., M. Saito, N. Oue, J. M. Weiss, J. Stauffer, S. Takenoshita, R. H. Wiltrout, S. P. Hussain and C. C. Harris (2013). "NOS2 enhances KRAS-induced lung carcinogenesis, inflammation and microRNA-21 expression." Int J Cancer **132**(1): 9-18.

Palayoor, S. T., J. B. Mitchell, D. Cerna, W. Degraff, M. John-Aryankalayil and C. N. Coleman (2008). "PX-478, an inhibitor of hypoxia-inducible factor-1alpha, enhances radiosensitivity of prostate carcinoma cells." Int J Cancer **123**(10): 2430-2437.

Puhakka, A., V. Kinnula, U. Napankangas, M. Saily, P. Koistinen, P. Paakko and Y. Soini (2003). "High expression of nitric oxide synthases is a favorable prognostic sign in non-small cell lung carcinoma." Apmis **111**(12): 1137-1146.

Puhakka, A. R., T. H. Harju, P. K. Paakko, Y. M. Soini and V. L. Kinnula (2006). "Nitric oxide synthases are associated with bronchial dysplasia." Lung Cancer **51**(3): 275-282.

Rattan, R., S. Giri, A. K. Singh and I. Singh (2005). "5-Aminoimidazole-4-carboxamide-1-beta-D-ribofuranoside inhibits cancer cell proliferation in vitro and in vivo via AMP-activated protein kinase." J Biol Chem **280**(47): 39582-39593.

Ravi, R., B. Mookerjee, Z. M. Bhujwalla, C. H. Sutter, D. Artemov, Q. Zeng, L. E. Dillehay, A. Madan, G. L. Semenza and A. Bedi (2000). "Regulation of tumor angiogenesis by p53-induced degradation of hypoxia-inducible factor 1alpha." Genes Dev **14**(1): 34-44.

Reisman, D. N., J. Sciarrotta, W. Wang, W. K. Funkhouser and B. E. Weissman (2003). "Loss of BRG1/BRM in human lung cancer cell lines and primary lung cancers: correlation with poor prognosis." Cancer Res **63**(3): 560-566.

Semenza, G. L. (2003). "Targeting HIF-1 for cancer therapy." Nat Rev Cancer **3**(10): 721-732.

So, K. S., J. K. Rho, Y. J. Choi, S. Y. Kim, C. M. Choi, Y. J. Chun and J. C. Lee (2015). "AKT/mTOR down-regulation by CX-4945, a CK2 inhibitor, promotes apoptosis in chemorefractory non-small cell lung cancer cells." Anticancer Res **35**(3): 1537-1542.

Tan, Y. H., S. Krishnaswamy, S. Nandi, R. Kanteti, S. Vora, K. Onel, R. Hasina, F. Y. Lo, E. El-Hashani, G. Cervantes, M. Robinson, H. S. Hsu, S. C. Kales, S. Lipkowitz, T. Karrison, M. Sattler, E. E. Vokes, Y. C. Wang and R. Salgia (2010). "CBL is frequently altered in lung cancers: its relationship to mutations in MET and EGFR tyrosine kinases." PLoS One **5**(1): e8972.

Tang, Y. C., B. R. Williams, J. J. Siegel and A. Amon (2011). "Identification of aneuploidy-selective antiproliferation compounds." Cell **144**(4): 499-512.

Wei, T. T., Y. C. Lin, P. H. Lin, J. Y. Shih, C. W. Chou, W. J. Huang, Y. C. Yang, P. W. Hsiao and C. C. Chen (2015). "Induction of c-Cbl contributes to anti-cancer effects of HDAC inhibitor in lung cancer." Oncotarget **6**(14): 12481-12492.

Welsh, S., R. Williams, L. Kirkpatrick, G. Paine-Murrieta and G. Powis (2004). "Antitumor activity and pharmacodynamic properties of PX-478, an inhibitor of hypoxia-inducible factor-1alpha." Mol Cancer Ther **3**(3): 233-244.

Zhang, S., H. Long, Y. L. Yang, Y. Wang, D. Hsieh, W. Li, A. Au, H. J. Stoppler, Z. Xu, D. M. Jablons and L. You (2013). "Inhibition of CK2alpha down-regulates Notch1 signalling in lung cancer cells." J Cell Mol Med **17**(7): 854-862.

Zhao, N., Y. Liu, Z. Chang, K. Li, R. Zhang, Y. Zhou, F. Qiu, X. Han and Y. Xu (2015). "Identification of Biomarker and Co-Regulatory Motifs in Lung Adenocarcinoma Based on Differential Interactions." PLoS One **10**(9): e0139165.
